# Can *Cis*-Regulatory Elements Explain Differences in *Petunia* Pollination Syndromes?

**DOI:** 10.3390/genes16080963

**Published:** 2025-08-15

**Authors:** Aléxia G. Pereira, João Pedro C. Filgueiras, Loreta B. Freitas

**Affiliations:** Department of Genetics, Universidade Federal do Rio Grande do Sul, 9500 Bento Gonçalves, Av., Porto Alegre 91509-900, Brazil

**Keywords:** transcription factors, *cis*-regulatory elements, *Petunia*, pollination syndromes

## Abstract

**Background**: Transcription factors have been linked to changes in various physiological processes, such as attractive and rewarding phenotypes during plant–pollinator interactions. In the genus *Petunia*, most species are pollinated by bees, but hawkmoth- and bird pollination are also observed. Here, we aimed to test the hypothesis that species with the same pollination syndrome evolved through convergence, while differences in pollinators indicate divergence. We selected six genes (*MYB-FL*, *DFR*, *EOBII*, *ODO1*, *BPBT*, and *NEC1*) involved in establishing pollination syndromes to explore the potential role of *cis*-regulatory elements in shifts among pollination syndromes, attracting and rewarding pollinators. **Methods**: We retrieved the genomic sequences of genes from the genomes of four *Petunia* species, which exhibit distinct pollination syndromes. We analyzed the *cis*-regulatory elements, focusing on the structure and composition of motifs, and inferred the functions of these transcription factors using Gene Ontology analysis. **Results**: All sequences were highly conserved among species, with variations in promoter motif structure and TF binding sites. The evolutionary relationships among the genes closely reflected the species’ phylogeny. Likewise, regulatory elements and gene structure mostly followed the species’ evolutionary history. However, different pollination syndromes are present, and there is an unexpected lack of convergence between the two bee-pollinated species. **Conclusions**: Our findings showed that the most recent common ancestor of these species better predicts relationships among gene regulatory elements than does the pollination syndrome. To fully understand the evolution of pollination syndromes in *Petunia*, additional studies are needed to analyze entire pathways and compare genomes and transcriptomes.

## 1. Introduction

*Cis*-regulatory elements are non-coding DNA sequences that regulate gene expression over time and across different tissues, which is essential for proper development and function of an organism. Additionally, changes in gene expression caused by alterations in *cis*-regulatory elements have been associated with the emergence of phenotypic novelties during evolution [1,2]. Transcription factors (TFs) control the expression of target genes by interacting with specific DNA elements, other TFs, and the basal transcription machinery. More than 1500 TFs have been identified in plants, regulating hundreds to thousands of genes [3]. Therefore, understanding and identifying *cis*-regulatory elements in genes is vital for uncovering the mechanisms behind phenotypic diversification.

With recent access to high-quality genomes, it becomes possible to conduct studies involving TF elements and infer their roles as parts of transcriptional networks for traits of interest, such as those involved in defining different pollination syndromes. Pollination syndromes describe groups of floral traits, such as general morphology, visible and ultraviolet light (UV) color, scent, and nectar production, that have evolved in response to the preferences, morphology, and behavior of specific pollinator groups. These floral trait combinations are shaped by pollinator-mediated selection and reflect adaptations to their sensory and ecological traits [4,5,6,7].

The interaction between flowering plants and their pollinators is a major factor driving the evolution and diversification of angiosperms [8]. The genus *Petunia*, part of the Solanaceae family, includes around 20 wild species [9] and the ornamental *P. hybrida*, a highly valued species widely used as a model in research [10,11]. Wild *Petunia* species are divided into two main groups based on the length of their corolla tubes. The short corolla tube group contains only bee-pollinated species, while the long corolla tube group includes species with three different pollination syndromes [9]. *P. inflata* shows ancestral features in its morphology and pollination syndrome [12]. This species has purple petals, a short corolla tube, emits no scent, reflects UV, and produces large amounts of concentrated nectar to reward pollinators [13]. The white-flowered *P. axillaris* is a relic of an “albino” lineage [14], which gave rise to all *Petunia* species with long corolla tubes [15]. This species attracts hawkmoths by releasing a strong, nocturnal scent [16] that is emitted by its UV-absorbing flowers [17], which provide nectar to insects [18]. *P. exserta* is pollinated by birds. It has bright red flowers, an elongated corolla tube, lacks a floral scent, exhibits UV reflectance, and produces nectar [13,19]. Additionally, another long corolla tube lineage, *P. secreta*, is bee-pollinated and features pink, scentless flowers that reward pollinators with abundant pollen [20]. All four species have sequenced genomes [10,21].

The transition between pollination syndromes in *Petunia* is well understood, and several speciation genes have been identified [16,17,21,22,23,24,25,26,27]. The pigmentation and UV reflectance of *Petunia* flowers are controlled by flavonoids, mainly anthocyanins and flavonols, which share di-hydroflavonols as common precursors [28]. TFs play a crucial role in regulating the balance between these pathways. In *P. axillaris*, the absence of anthocyanins, caused by inactivation of a *R2R3 MYB* (*MYB-FL*) TF that activates di-hydroflavonol-4-reductase (*DFR*), results in white corollas [29]. Conversely, in *P. secreta*, the reactivation of *MYB* restores purple pigmentation [26]. The red color of *P. exserta* is controlled by a complex regulatory network that modulates cyanidin and delphinidin levels, despite the *DFR* gene being inefficient at processing red anthocyanin precursors [21,30]. Flavonol accumulation and UV response are regulated by *MYB-FL*, which activates flavonol synthase [25]. In *P. axillaris*, the positive regulation of *MYB-FL* leads to high UV absorption, whereas gene inactivation in *P. exserta* and repression in *P. secreta* cause UV-reflective corollas due to reduced flavonoid levels [25,26]. Variations in *MYB-FL* expression among species are linked to *cis*-regulatory mutations within or near enhancer or promoter regions [26].

The emission of floral scent is controlled by the floral volatile benzenoid/phenylpropanoid pathway, primarily regulated by *ODORANT1* (*ODO1*) gene, a key activator of downstream genes [31,32]. The TFs *EMISSION OF BENZOIDS I AND II* (*EOBI* and *EOBII*) increase *ODO1* expression at night, while *LATE ELONGATED HYPOCOTYL* gene suppresses it during the day [33,34,35]. In *P. exserta*, the shift from hawkmoth to hummingbird pollination is associated with the decreased *ODO1* expression and inactivation of cinnamate: CoA ligase, resulting in scent loss [17]. Although *P. secreta* flowers are also scentless [36], pollen-derived volatiles have been shown to attract bees [20]. In *P. inflata*, another bee-pollinated species, volatile biosynthesis is suppressed due to the inactivation of late-pathway genes such as benzoyl-CoA: benzylalcohol/2-phenylethanol benzoyltransferase (*BPBT*) and S-adenosyl-L-methionine: benzoic acid/salicylic acid carboxyl methyltransferase [17], and this species provides only nectar as a reward to pollinators.

The *NECTARY 1* (*NEC1*) protein functions as a nectar-specific sugar transporter, essential for nectar production, which affects both its concentration and viscosity [37]. In *P. hybrida*, *NEC1* is expressed only in nectaries and affects mainly nectar production [38]. Mainly characterized in *P. hybrida*, *NEC1* is an ortholog of SWEET9c in *P. axillaris*, a gene that displays a similar expression pattern [39].

Several studies have explored the function, regulation, and sequences of key genes involved in the anthocyanin, flavonol, and floral volatile benzenoid/phenylpropanoid (*FVBP*) pathways in both corolla and pollen tissues, as well as genes related to nectar sugar content. In *Petunia*, the quick evolutionary radiation among species has led to low divergence in coding sequences, raising questions about the role of *cis*-elements in phenotypic differences. Here, we examined the promoter regions of genes involved in pathways that attract or reward pollinators in *Petunia* species. We tested the hypothesis that differences in pollination syndromes among *Petunia* species are associated with variations in the *cis*-regulatory elements of key floral genes, with convergence among species sharing the same pollinator functional group and divergence when they differ in pollination syndrome.

## 2. Materials and Methods

### 2.1. Genes

**Figure 1:**
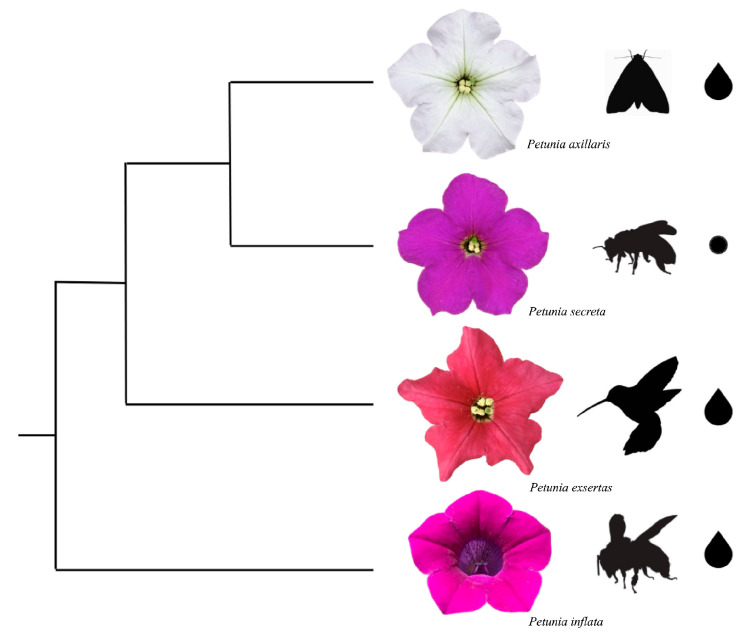
Evolutionary relationships among *Petunia* species, with flowers shown in frontal view to highlight visible color phenotypes, their respective pollinators, and sources of rewards (drops indicate nectar and dots indicate pollen). From top to bottom, *P. axillaris* and *Manduca sexta*; *P. secreta* and *Pseudagapostemon* sp.; *P. exserta* and an unidentified hummingbird; *P. inflata* and *Hexantheda* sp. Photos are from the authors’ collection.

We selected six genes involved in floral attraction and rewards for pollinators: the transcription factor *MYB-FL*, which responds to UV; the *DFR* gene, which functions in the anthocyanin pathway; *ODO1*, *EOBII*, and *BPBT* genes, related to scent emission and composition; and *NEC1*, a sugar efflux transporter that contributes to nectar sugar concentration and composition. These genes were selected to characterize the differential phenotypes in *P. inflata*, *P. axillaris*, *P. secreta*, and *P. exserta*, which have diverse pollinator syndromes (Figure 1).

### 2.2. Gene and Protein Sequences

We retrieved *Petunia* *BPBT* and *EOBII* genomic sequences from Sol Genomics Network (https://solgenomics.net/; accessed on 13 August 2025), National Center for Biotechnology Information (*NCBI*; https://www.ncbi.nlm.nih.gov/; accessed on 13 August 2025), and DNAZoo (https://www.dnazoo.org/assemblies/Petunia_exserta; accessed on 13 August 2025) following a previously published protocol [40].

Additionally, we used the already published sequences of the *DFR*, *ODO1*, *MYB-FL*, and *NEC1* genes [40], except for *P. secreta* *NEC1*, which was previously reported as incomplete despite the species producing nectar [20]. For *P. secreta*, we searched for *NEC1* in the new genome version available at the National Center for Biotechnology Information (https://www.ncbi.nlm.nih.gov/datasets/taxonomy/323111/; accessed on 13 August 2025) using *P. axillaris NEC1* as a query.

Initially, we compared protein sequences by translating the CDS into amino acid sequences using MEGA11 [41]. Then, we created multiple alignments for each gene with GenomeNet Sequence Analysis CLUSTALW (https://www.genome.jp/tools-bin/clustalw; accessed on 13 August 2025) using default settings and highlighted conserved amino acids with a BOXSHADE analysis (https://junli.netlify.app/apps/boxshade/; accessed on 13 August 2025).

### 2.3. Promoter and Gene Structure Analyses

We recovered a 2 kb segment upstream of the translation start site for each gene (Supplementary Table S1). We performed TF binding site prediction using PlantRegMap (http://plantregmap.gao-lab.org/binding_site_prediction.php; accessed on 13 August 2025) for *Arabidopsis thaliana* and *Solanum lycopersicum* (threshold *p*-value ≤ 10^−5^). We only retained TF binding predictions on the transcribed DNA strand in the negative direction. We used TBtools-II [42] to create the visual models of TF binding sites in promoters. These models were edited with Inkscape v.1.3.2 (https://inkscape.org/pt-br/; accessed on 13 August 2025), a free and open-source vector graphics editor.

We examined conserved motifs in each promoter using MEME v.5.3.3 (http://meme-suite.org/tools/meme; accessed on 13 August 2025), allowing for 12 motifs with lengths ranging from 6 to 110 nucleotides. The motifs were numbered starting at the ATG at the 3′ end. Additionally, we utilized the FIMO tool in the MEME Suite (https://meme-suite.org/meme/tools/fimo; accessed on 13 August 2025) to search for the previously described *cis*-elements in *ODO1* [35,43], *BPBT* [32], and *NEC1* [35] using the default settings.

### 2.4. Gene Ontology

We performed a GO enrichment analysis using Gene Ontology (https://geneontology.org; accessed on 13 August 2025), with the access code from PlantRegMap, powered by PANTHER [44], to investigate the potential functions of the TF families associated with the promoters of the genes of interest. We used the default mode based on *A. thaliana* and filtered by biological processes. We summarized the list of GO identifiers in Revigo (http://revigo.irb.hr; accessed on 13 August 2025) using the default settings, and removed redundant GO terms. The results were visualized as Venn diagrams to highlight the similarities and differences between GO terms across species and genes.

## 3. Results

### 3.1. Gene and Protein Sequences

**Figure 2:**
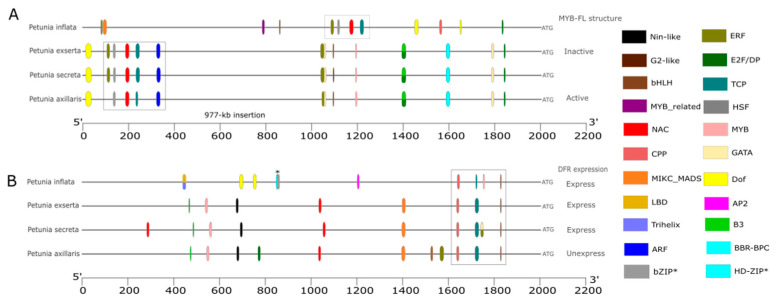
TF binding sites identified in the promoter regions of the color pathway genes *MYB-FL* (**A**) and *DFR* (**B**), based on PlantRegMap analysis using *A. thaliana* as the reference. *MYB-FL* is a TF that activates *FLS*, promoting flavonol production, while *DFR* is the first gene in the anthocyanin branch; both share the precursor dihydroflavonol. Activation of one pathway can limit the other due to competition for this common substrate. Each color represents a TF, as indicated by the legend on the right. Multiple colors indicate overlapping TFs. Asterisks mark sites identified with the same color.

All protein sequences showed high conservation among *Petunia* species (Supplementary Figures S1–S6), with variations in promoter motif structure and TF binding sites. The TF binding prediction against *A. thaliana* generally identified more *cis*-elements than when using *S. lycopersicum* as a reference. Compared to *P. inflata*, we found a 977 bp insertion in the *MYB-FL* promoter of *P. axillaris*, *P. secreta*, and *P. exserta*, which corresponds to motifs three to eight (Supplementary Figure S7), changing the organization of TF binding sites between *P. inflata* and the remaining *Petunia* species (Figure 2A and Supplementary Figure S8A). All *Petunia* species shared an E2F binding site near the start codon (ATG site) and a signaling cluster, including TCP, NAC, HSF, and ERF (indicated by a square in Figure 2A), except *P. axillaris*, which lacks the ERF binding site. This signaling cluster is closer to the start codon in *P. inflata* than in the other three species due to the insertion found in the long corolla tube species. Additionally, species with long corolla tubes have a DOF binding site before these TF binding sites. Most TF binding sites were conserved among *P. axillaris*, *P. secreta*, and *P. exserta*, except for one ERF site missing in *P. axillaris*. In the analysis using *S. lycopersicum* as a reference (Supplementary Figure S8A), we also observed that *P. inflata* and other *Petunia* species showed greater divergence in TF binding site composition. Regarding the signaling cluster, it was characterized only by TCP and HSF binding sites. In the long corolla tube species, there was an increase in overlapping binding sites, especially between ERF and GATA (1030 bp), *MYB* and ERF (1200 bp), and C2H2 and BBR-BPC (1600 bp).

For the *DFR* promoter, *P. inflata* has a deletion corresponding to motifs 8 to 12 (Supplementary Figure S9) when compared to species with a long corolla tube. TF binding sites (Figure 2B) for *MYB*, *bHLH*, *TCP*, and *CPP* families were found in all four species. All *cis*-elements observed in *P. exserta* were also present in *P. axillaris* and *P. secreta*. However, two species had three additional sites, *P. secreta* (NAC, GATA, and E2F/DP) and *P. axillaris* (ERF, bHLH, and E2F/DP). Using *S. lycopersicum* as a reference (Supplementary Figure S8B) revealed some differences. For example, bHLH binding sites are present in *P. secreta* and *P. exserta*, while an extra NAC binding site appears in *P. exserta* and *P. axillaris*. The *A. thaliana* database analysis did not show these binding sites (Figure 2B).

**Figure 3:**
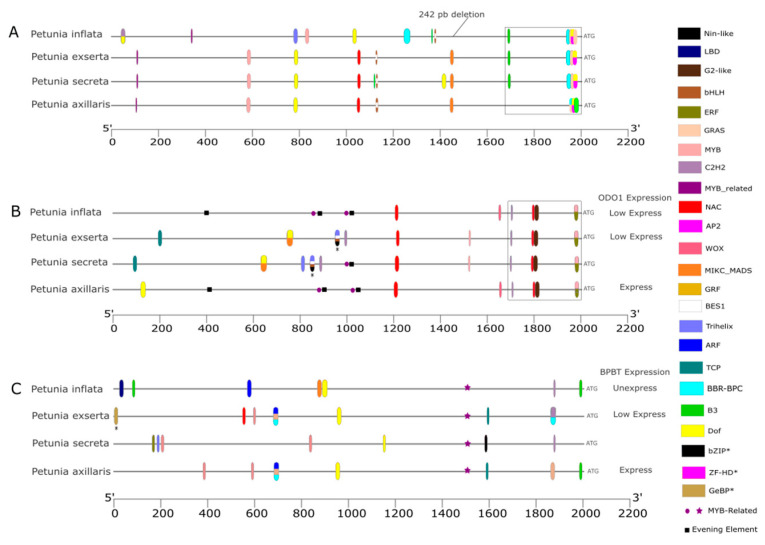
TF binding sites identified in the promoter regions of the scent pathway (*FVBP*) genes *EOBII* (**A**), *ODO1* (**B**), and *BPBT* (**C**), based on PlantRegMap analysis using *A. thaliana* as a reference. *EOBII* acts upstream of *ODO1* and other scent-related genes, promoting its expression at night. *LHY* represses *ODO1* expression during the day, aligning volatile emission with the circadian cycle. *ODO1* drives *BPBT* expression, leading to the production of the final volatile compound, phenyl ethyl benzoate. Each color represents a TF, as indicated in the legend on the right. Multiple colors indicate overlapping TFs. Asterisks denote sites identified with the same color. Purple dots indicate the binding site of *EOBII* (AAACCTAAT), and black squares indicate *LHY* (AAAATATCT) found in the *ODO1* promoter using FIMO. Purple stars mark an *ODO1* binding site (CAACAACTAC) observed in the *BPBT* promoter.

Regarding the *EOBII* protein sequence (Supplementary Figure S10), there are no differences among long corolla tube species, whereas *P. inflata* has lost six motifs (motifs three to eight). The *P. inflata EOBII* promoter, compared to other *Petunia* species (Supplementary Figure S11), lost a 242 bp segment that corresponds to motif four, a MICK-MADS TF binding site (Figure 3A and Supplementary Figure S12A). The species *P. secreta* and *P. exserta* were most similar in the composition and positioning of TF-binding sites, with only two additional sites in *P. secreta.* Only two sites were conserved among the four species, although in different positions in *P. inflata* (overlapped *bHLH*/*BES1*). Near the ATG site, a conserved signaling cluster (indicated by a square in Figure 3A) is present, which varies in position among the species. The *P. secreta* and *P. exserta* clusters are identical in composition to *P. inflata*, which has a duplicated GRAS. In *P. axillaris*, this cluster includes an additional B3 and an overlap between BBR-BPC and AP2. The analysis using *S. lycopersicum* as a reference revealed increased complexity in the conserved signaling cluster due to overlapping TF binding sites. The position of the B3 binding sites in *P. axillaris* was shifted to 1700 bp. An additional C2H2 binding site was identified in all *Petunia* species (Supplementary Figure S12A).

In the *ODO1* promoter (Supplementary Figure S13), motif seven was duplicated in *P. secreta* and *P. exserta*. The other motifs remained conserved among species, with variations in their positions relative to motifs 11 and 12. TF binding sites (Figure 3B) were conserved in both composition and position between *P. axillaris* and *P. inflata*, except for an additional DOF in *P. axillaris*. The TF binding site composition was similar between *P. secreta* and *P. exserta*, except that *P. secreta* had extra Trihelix- and *MYB*-related sites. In the analysis using *S. lycopersicum* as a reference (Supplementary Figure S12B), the NAC- and G2-like sites at the 1800 bp position were not found in all *Petunia* species. Instead, a BBR-BPC was identified. This also suggests a greater similarity in the predicted TF binding motifs in the promoters of *P. exserta* and *P. secreta*.

The four *Petunia* species conserved the 12 motifs in *BPBT* protein (Supplementary Figure S14). The MEME analysis of the *BPBT* promoter (Supplementary Figure S15) showed the same motif composition for *P. axillaris*, *P. secreta*, and *P. exserta*, with the only difference being the position of motif nine in *P. secreta*. *P. inflata* lacked motif eight. Regarding TF binding sites, the four *Petunia* species showed variations, with different compositions and positions for these elements (Figure 3C). When analyzing *BPBT* using *S. lycopersicum* as a reference (Supplementary Figure S12C), notable differences in binding site identification were observed compared to the results using *A. thaliana* as a reference. At 580 bp position in *P. inflata*, a shift from ARF to TALE was observed. In *P. exserta* and *P. axillaris*, a transition from DOF to LBD occurred at 900 bp. Additionally, in *P. axillaris*, GRAS was replaced with GRF at 1900 bp. Both analyses reveal greater similarity between the promoters of *P. exserta* and *P. axillaris* compared to the other two species, although differences in the predicted TF binding motifs remain.

**Figure 4:**
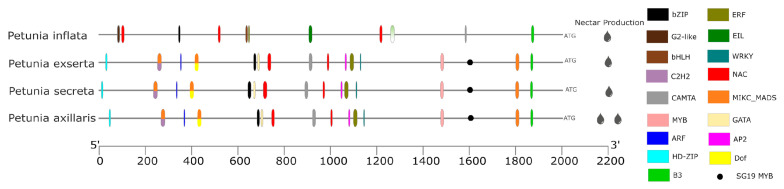
TF binding sites identified in the promoter regions of the sugar efflux transporter gene *NEC1* based on PlantRegMap analysis using *A. thaliana* as a reference. Each color represents a TF, as shown in the legend on the right. Multiple colors indicate overlapping TFs. Asterisks mark sites identified with the same color. The black dot shows the binding site for *EOBII* (GTTTGGT), as identified with FIMO.

The *NEC1* promoter was conserved among *P. axillaris*, *P. secreta*, and *P. exserta* in terms of motifs (Supplementary Figure S16) and predicted TF binding sites (Figure 4), while *P. inflata* was completely different in composition and structure, losing nine motifs and displaying motif two duplicated. In the analysis of *NEC1* using *S. lycopersicum* as a reference (Supplementary Figure S17), discrepancies in TF binding site identification were found compared to the results using *A. thaliana* as a reference. In *P. inflata*, a substitution of TALE and C2H2 for EIL was observed at 900 bp, along with a transition from CAMTA to SBB at 1650 bp. In *P. axillaris*, *P. secreta*, and *P. inflata*, changes in binding sites at 900 bp were also detected, with a transition involving LBD and CAMTA to CAMTA alone.

The search for previously published *cis*-regulatory sequences identified two *MYB* binding sites for *EOBII* (AAACCTAAT) in the *ODO1* promoter of *P. inflata* and *P. axillaris*, and one in *P. secreta*. These sites were classified as *MYB*-related based on PlantRegMap analysis (purple dots in Figure 3B and Supplementary Figure S8A). Additionally, an *LHY cis*-element (AAAATATCT) was found, which was triplicated in *P. axillaris* and *P. inflata* but single-copy in *P. secreta* (black squares in Figure 3B and Supplementary Figure S12B). FIMO analysis revealed *EOBII* and *LHY* sites in the *ODO1* promoter that PlantRegMap did not detect (Supplementary Table S2). In the *BPBT* promoter, a binding site for *ODO1* (CAACAACTAC) was found in all *Petunia* species (purple star in Figure 3C and Supplementary Figure S12C), and along with *DOF*, it was the only *BPBT cis*-element common to these species. FIMO analysis also identified a *cis*-regulatory sequence for *EOBII* (GTTTGGT) in *NEC1* of *P. axillaris*, *P. secreta*, and *P. exserta* (black dots in Figure 4 and Supplementary Figure S17).

### 3.2. Gene Ontology

Based on the GO analysis (Supplementary Table S3), we observed a varying number of predicted transcription factor binding sites (using *A. thaliana* as a reference) involved in different cellular processes per gene and per species. Concerning the response to visible and UV light, we identified 62 predicted TF binding sites for *MYB-FL*, of which 13 were shared among the four *Petunia* species (Supplementary Figure S18A). These TFs were related to 37 GO terms (Supplementary Figure S18B) involved in regulating biological processes, including development and DNA transcription. The long corolla tube species shared 33 predicted TF binding sites (*ERF*, *MYB*, *GATA*, *DOF*, *BBC-BPC*, *E2F/DP*, and *ARF* families), which participate in abiotic stress responses and hormonal signaling. *P. exserta* and *P. secreta* share six *MYBs* associated with the regulation of lipid synthesis, and responses to heat, drought, and oxygenated compounds. In contrast, seven predicted TF-binding sites are unique to *P. inflata* (*MYB*-related, *DOF*, *MICK-MADS*, and B3 families), which are involved in regulating the circadian rhythm, leaf senescence, and nucleosome organization.

In the *DFR* promoter (Supplementary Figure S18C), we identified 18 predicted TF binding sites common to all *Petunia* species, including *bHLH*, *TPC*, and *CPP*. These TFs are associated with 18 GO terms (Supplementary Figure S18D) related to DNA transcription and anthocyanin biosynthesis. Additionally, *P. secreta* has 11 unique predicted TF binding sites (ERF and GATA families), which respond to water and are involved in the breakdown of terpenoids. Moreover, the TFs in *P. axillaris* play roles in cell division, root development, and signal transduction.

Regarding aroma production, *Petunia* species exhibited 23 predicted TF-binding sites in the *EOBII* gene promoter (Supplementary Figure S19A), most of which belong to the bHLH family. These TFs are associated with 34 GO terms (Supplementary Figure S19B) involved in various biological processes, including responses to endogenous stimuli, plant organ development, DNA transcription, and the biosynthesis of flavonols and anthocyanins. In *P. inflata*, two unique predicted TF binding sites (Trihelix and *C2H2*) were identified as involved in responses to jasmonic acid, regulation of fatty acid biosynthesis, and mediation of gibberellin signaling pathways. In the *ODO1* gene promoter, *Petunia* species share eight predicted TF binding sites (*NAC*, *ERF*, *G2-like*, *C2H2*, *BBR-BPC*, and *MYB* families), but these TFs do not have the same functions in GO (Supplementary Figure S19C,D). *P. exserta* and *P. secreta* share a set of 27 predicted TF binding sites, related to responses to red light, cellular and biological regulation, responses to endogenous stimuli, and regulation of DNA transcription. *P. secreta* and *P. exserta* show species-specific predicted TF binding sites for the *ODO1* promoters; in *P. secreta*, the TF binding site relates to hormones, while in *P. exserta*, it is linked to abscisic acid and alcohol.

The promoters of the *BPBT* gene differ in composition and structure, with each species exhibiting specific predicted TF binding sites (Supplementary Figure S19E,F). *Petunia secreta* does not share any predicted TF binding site with the other species. The nine commonly predicted TF binding sites (*TPC*, *BBR-BPC*, *MYB*, *AP2*, and *GRAS* families) between *P. axillaris* and *P. exserta* are associated with GO terms related to fundamental functions in regulating cellular, metabolic, and biological processes. A unique predicted TF-binding site is shared between *P. axillaris* and *P. inflata.* It is associated with ethylene response and the regulation of nucleobase metabolism in *P. axillaris*, and with the positive regulation of cellular processes in *P. inflata*.

We identified three shared predicted TF binding sites (NAC and B3 families) related to seven GO in *NEC1* (Supplementary Figure S20A,B). The GOs are involved in fundamental developmental functions. Among long corolla tube species, 31 predicted TF binding sites are shared and linked to tissue development, responses to endogenous stimuli, and anther dehiscence. In *P. inflata*, the 27 exclusive TF binding sites are associated with the differentiation of tracheary elements, which are specialized cells that conduct sap and form part of the xylem.

## 4. Discussion

The genus *Petunia* is a relatively young group of plants with species divided into two main groups. Each group is supported by the length of the corolla tube [9]. *P. axillaris*, *P. exserta*, and *P. secreta* have long corolla tubes, while *P. inflata* belongs to the short corolla tube group. The evolutionary relationships among gene sequences involved in pollinator-attractive traits closely reflect the species’ phylogeny, consistent with other studies based on Solanaceae genes and processes [40,45,46,47]. Likewise, regulatory elements and gene structures mostly follow the species’ evolutionary history. However, different pollination syndromes are present, and there is an unexpected lack of convergence between the two bee-pollinated species.

The observed similarities among species with long corolla tubes and different pollinators may stem from their common ancestry. The white *P. axillaris* is at the base of this group, with *P. exserta* and *P. secreta* as the more recently evolved species [15]. Divergence among these species has been associated with local adaptation, which enables them to attract new pollinators or colonize new environments [48,49,50].

The *MYB-FL* regulatory regions diverged between the two clades, with variations in *cis*-element composition, and placement between *P. inflata* and the long corolla species. The structure of the *MYB-FL* gene remained consistent across *P. axillaris*, *P. exserta*, *P. secreta*, and *P. inflata.* However, levels of UV-absorbing floral flavonols vary among these species. *P. axillaris* differs from the other species in the clade by only one *ERF* receptor. *EFR* plays a crucial role in responses to biotic and abiotic stresses, acting as an activator or repressor in various pathways involved in anthocyanin and flavonol biosynthesis [51]. Flavonols may also play a role in stress responses [52]. The species in the long corolla tube clade differ in microenvironment [15], with *P. secreta* and *P. exserta* occupying drier habitats than *P. axillaris*, which may be linked to GO terms related to temperature and drought response.

Based on the genus phylogeny, bee pollination is likely the ancestral state, with hawkmoths and hummingbirds as the derived pollination states [12]. This hypothesis assumes that ancestral flowers were UV-reflective and exhibited colors such as pink or purple [14]. The recently proposed phylogeny of the long corolla tube clade indicates that all species in this group descended from an “albino” ancestor (white flowers and yellow pollen), probably a *P. axillaris*-like lineage [15]. The transition from bee to hawkmoth pollination (from *P. inflata*-type to *P. axillaris*-type) involves increased UV absorbance due to the upregulation of the *MYB-FL* promoter. Simultaneously, a frameshift mutation in the *MYB-FL* gene influences the shift from hawkmoth to hummingbird pollination (from *P. axillaris* to *P. exserta*) [25]. A similar pattern likely contributed to the UV response in *P. secreta*, which reflects UV light [26], despite this species being a descendant of a *P. axillaris*-like lineage [15].

The white color in *P. axillaris* results from the absence of anthocyanin caused by the inactivation of *AN2*. This transcription factor activates the *DFR* gene and other genes involved in anthocyanin biosynthesis [29]. The pink color seen in the corolla limb of *P. secreta* is due to the reactivation of *AN2* caused by a single mutation that restores the reading frame [26]. The red color in *P. exserta* is more complex because the *DFR* gene does not recognize dihydrokaempferol, a precursor of the red anthocyanin [30], and *AN2* is non-functional. However, the paralog of *AN2*, *DPL*, restores anthocyanin biosynthesis. This, along with other genes, creates the red color by balancing cyanidin (pink) and delphinidin (purple) anthocyanin precursors [21].

The anthocyanin biosynthesis process starts with multiple steps that convert dihydroflavonol, catalyzed by several enzymes including *DFR*, which produces unstable leucoanthocyanidins [53]. The structure of *DFR* was conserved across both short and long corolla tube groups, mainly in the epimerase domain [40], although regulatory elements varied. *Cis*-elements differed in number, location, and type, even among species with long corolla tubes. This supports the idea that *DFR* plays a secondary role in shaping visible color in *Petunia*, which is mainly controlled by *AN2* in *P. axillaris* (white), *P. secreta* (pink), and *P. inflata* (purple) [26], as well as by a complex network of small-effect genes in *P. exserta* [21]. Environmental factors often influence changes in corolla hue. The roles of transcription factors that bind to *DFR* promoters suggest that different stimuli, including hormonal signals, responses to nitrogen availability, and water stress, can influence their expression. The buildup of visible pigmentation depends on *DFR* expression, which drives the conversion of precursors into anthocyanins. Therefore, the intensity of corolla coloration may be linked to environmental and physiological factors that regulate *DFR* expression.

The biosynthesis and release of floral volatiles depend on the coordinated regulation of various enzymes and compounds. The genes *EOBII* and *ODO1* are involved early in this process [31,43]. In *Petunia* species, *BPBT* plays a crucial role in producing and releasing aromas [17]. The scentless *P. inflata* differs from the fragrant *P. axillaris* in the composition of the *EOBII* motifs. However, the gene structure remains conserved in species with long corolla tubes, even though *P. secreta* and *P. exserta* are described as scentless [13]. Some transcription factors predicted to bind the *EOBII* promoter have similar roles, which suggests that this gene may be involved in pathways beyond volatile compound biosynthesis [35]. The *ODO1* structure and protein motif composition follow the *EOBII* pattern; however, the regulatory elements exhibit significant divergence. The promoter region of scented *P. axillaris* is more similar to that of the unscented *P. inflata* than to other long corolla tube species, with only one additional *DOF* site (Figure 3B). We found that *P. exserta* has lost all predicted TF binding sites for *EOBII* and *LHY*, which regulate the activation of *ODO1* in the evening and its circadian repression in the morning, respectively. This likely contributes to the low expression of *ODO1* and reduced scent emission in *P. exserta* [17]. Since *ODO1* is a key TF that activates multiple genes in the *FVBP* pathway, its decreased expression may limit the biosynthesis of floral volatile compounds, explaining the scentless phenotype of *P. exserta* flowers. Similarly, our analysis showed that *P. secreta* has partially lost the binding sites for *EOBII* and *LHY*. Although *ODO1* expression in *P. secreta* has not yet been characterized, we hypothesize it may also be downregulated, leading to reduced floral volatile emission. While *P. secreta* is described as scentless, volatile compounds have been identified in its pollen [20], which is used as a floral reward to attract its specific pollinator.

*BPBT* showed no variation in protein structure among *Petunia* species. However, *cis*-regulatory elements in this gene differed among these species, indicating a notable divergence in transcriptional activation, regardless of whether the species is scented or unscented. Scent emission in flowers depends on the availability of precursor compounds produced by enzymatic reactions across specific metabolic pathways. In *Petunia*, *ODO1* influences the expression of early genes in the *FVBP* pathway, while *EOBII* controls both *ODO1* and additional downstream genes. Furthermore, *EOBII*, *ODO1*, and *BPBT* are regulated by the circadian clock, showing distinct expression patterns between *P. axillaris* and *P. integrifolia* [34]. *Petunia integrifolia*, another species with a short corolla tube and no scent, is closely related to *P. inflata* [9].

The floral scent is crucial for *P. axillaris* pollination because the specific pollinator, *M. sexta*, is preferentially attracted to aroma rather than any other cue [17]. Other *Petunia* species do not rely on scent emission, as hummingbirds use gustatory, tactile, and visual cues to find food and avoid dangers [54]. In contrast, bees use nectar guides, the visible color of petals, or even pollen aroma, as observed in *P. secreta* [20].

The lack of convergence in gene and regulatory element analyses between the two bee-pollinated species, *P. inflata* and *P. secreta*, may partly stem from differences in the behavior of their pollinators. *P. inflata* is pollinated by bees of the genus *Hexantheda* [55], which are attracted to the blue pollen and nectar and often use the flowers as a dormitory. In contrast, *Pseudagapostemon* bees are attracted to *P. secreta* flowers by their distinctive yellow pollen aroma, which serves as the primary reward for the pollinators [20].

Floral nectar is a sugary, complex, water-based solution mainly made up of three simple sugars: sucrose, fructose, and glucose. These sugars come from the same precursor and processes [56,57], but their levels vary widely. The amount of sugar differs among plant species and depends on the primary pollinator involved [58]. While moth- and bird-pollinated plants typically produce large amounts of dilute nectar, bee-pollinated species usually produce smaller quantities of more concentrated nectar, as observed in *P. inflata* [13]. As *Pseudagapostemon* bees do not reach the nectar in *P. secreta* [20], since the nectary chamber is inaccessible to them, the nectar composition and other characteristics are free to change in this species.

The long corolla tube of *Petunia* species did not differ in nectar volume and sugar concentration (Pereira et al., unpublished data), despite their differing pollinator preferences. Furthermore, the nectar in these species is only accessible to long-tongued pollinators because the nectaries are positioned in a ring surrounding the base of the ovary wall [59]. Among the long corolla tube species, which share traits related to nectar characteristics and their genetic basis, this could be due to their most recent common ancestor. This indicates a factor other than pollinator-driven selection. In contrast, *P. inflata* produces small amounts of nectar with high sugar concentrations [60]. Once again, neither the structure of the analyzed genes [40] nor their regulatory elements are enough to explain the divergent pollination syndromes in these *Petunia* species.

The initial description of the *NEC1* gene indicated that its expression was observed in both the parenchyma cells of the nectary and the upper part of the filament, as well as in the stoma of the anther [38]. Additionally, transgenic plants showed leaves with three-to-four times more phloem bundles in the veins compared to the wild type. These findings are related to the GO associated with the TFs of the *NEC1* promoter, including anther dehiscence, differentiation of tracheal elements (cells specialized in sap conduction), and tissue development.

While the in silico approach used here with PlantRegMap allowed us to identify potential transcription factor binding sites in the promoter regions of target genes, some methodological limitations should be acknowledged. First, the predictions rely on known *cis*-regulatory elements from *A. thaliana* and *S. lycopersicum*, which may not fully reflect the regulatory structure of *Petunia* species due to evolutionary differences. Additionally, binding site predictions do not confirm actual TF binding or gene regulation in living organisms. Therefore, experimental validation, such as ChIP-seq, reporter assays, or expression studies involving TF overexpression or knockdown, is necessary to verify these interactions. The analysis was also limited to 2000 bp upstream of the start codon, potentially missing distant enhancers or other regulatory elements. Furthermore, although we interpreted differences in TF binding site composition across species in relation to the floral phenotype and pollinator attraction, other regulatory layers, such as epigenetic modifications, chromatin accessibility, or post-transcriptional regulation, were not examined and may also influence the observed differences.

## 5. Conclusions

In conclusion, our results underscore the strong phylogenetic basis for promoter conservation and shared polymorphisms between the long corolla tube clade and its divergence from *P. inflata*, which represents the short corolla tube clade in the genus *Petunia*. Despite differences in pollination syndromes, *P*. *axillaris*, *P. exserta*, and *P. secreta* exhibit conserved promoter regions in key regulatory genes such as *MYB-FL*, *DFR*, *EOBII*, and *NEC1*, indicating a deep ancestral regulatory framework.

Interestingly, the promoter similarity of *ODO1* was observed between the scented *P. axillaris* and the scentless *P. inflata*. At the same time, the most significant divergence occurred between *P. secreta* and *P. exserta*, both of which are scentless but use different pollination strategies. Notably, *P. exserta* has lost all predicted TF-binding sites for *EOBII* and *LHY*, whereas *P. secreta* has experienced a partial loss, which may explain their reduced *ODO1* expression and scentless floral traits. This separation of gene regulation from pollination strategy highlights the complex nature of floral trait evolution. In contrast, the *BPBT* promoter showed distinct compositions across all species, regardless of scent production, suggesting lineage-specific regulation or varying selective pressures.

Although analyses using the *S. lycopersicum* database identified fewer TF binding sites than those based on *A. thaliana*, likely due to differences in functional studies, both methods generally agree on core *cis*-elements and TF families, despite their low frequency. GO annotations mainly relate to broad regulatory functions, such as controlling biological processes and DNA transcription, with occasional gene- or species-specific enrichments indicating functional divergence.

Our findings reveal a complex and intricate network that determines phenotypes by attracting and rewarding pollinators, with no convergence observed between species sharing the same functional pollinator group, such as *P. secreta* and *P. inflata*. Conversely, species pollinated by different functional groups, such as *P. axillaris*, *P. exserta*, and *P. secreta*, share similar gene structures and regulatory elements. To fully understand the genetic basis of pollination syndrome evolution in *Petunia*, future studies should combine whole-genome and transcriptome comparisons with functional assays of promoter activity across various developmental stages and environmental conditions.

## Data Availability

All datasets generated during the current study are included in the main text or available in the online  Supplementary Materials.

## Supplementary Materials

The following supporting information can be downloaded at https://www.mdpi.com/article/10.3390/genes16080963/s1, Figure S1: Protein box shading for *MYB-FL* alignment; Figure S2: Protein box shading for *DFR* alignment; Figure S3: Protein box shading for *EOBII* alignment; Figure S4: Protein box shading for *ODO1* alignment; Figure S5: Protein box shading for *BPBT* alignment; Figure S6: Protein box shading for *NEC1* alignment; Figure S7: *MYB-FL* promoter conserved motifs identified through MEME analysis; Figure S8: TF binding sites identified in the promoter regions of the color pathway genes with PlantRegMap analysis referencing *S. lycopersicum*; Figure S9: *DFR* promoter conserved motifs identified through MEME analysis; Figure S10: *EOBII* protein conserved motifs identified in MEME analysis; Figure S11: *EOBII* promoter conserved motifs identified by MEME analysis; Figure S12: Transcription factor binding sites identified in the promoter regions of the scent pathway (*FVBP*) genes based on PlantRegMap analysis using *S. lycopersicum* as the reference; Figure S13: Conserved motifs in the *ODO1* promoter identified by MEME analysis; Figure S14: *BPBT* protein conserved motifs identified by MEME analysis; Figure S15: *BPBT* promoter conserved motifs identified in MEME analysis; Figure S16: Conserved motifs in the *NEC1* promoter identified through MEME analysis; Figure S17: Transcription factor binding sites identified in the promoter regions of the sugar efflux transporter gene based on PlantRegMap analysis using *S. lycopersicum* as a reference; Figure S18: Venn diagrams illustrating genes involved in pigment biosynthesis pathways; Figure S19: Venn diagrams illustrating genes involved in volatile biosynthesis pathways; Figure S20: Venn diagrams for the *NEC1* gene; Table S1: Genome code for recovered sequences per gene and species; Table S2: *Cis*-regulatory elements in *ODO1*, *BPBT*, and *NEC1* genes were revealed using the FIMO tool in the MEME suite [61]; Table S3: List of Gene Ontology (GO) identifiers summarized by Revigo.

## Abbreviations

The following abbreviations are used in this manuscript:

bp	base pairs
BPBT	benzoyl-CoA: benzyl alcohol/phenyl ethanol benzoyl transferase gene
CDS	coding DNA sequence
DFR	di-hidroflavonol-4-reductase gene
EOBII	emission of benzoids II gene
FVBP	floral volatile benzenoid/phenylpropanoid
GO	Gene Ontology analysis and terms
kb	kilo bases
MYB-FL	R2R3 MYB transcription factor
NEC1	nectar 1 gene
ODO1	odorant 1 gene
TF-binding	transcription factor binding sites
TF(s)	transcription factor(s)
UV	ultraviolet
