# Supplementary material for: Can Cis-Regulatory Elements Explain Differences in Petunia Pollination Syndromes?
Source: Genes (Basel). 2025 Aug 15;16(8):963. doi: 10.3390/genes16080963 (PMC12385822; doi:10.3390/genes16080963)
Supplement: Supplementary file 1 [file genes-16-00963-s001.zip › genes-3805741-supplementary.pdf]

Article

Can *cis*-regulatory elements explain differences in *Petunia* pollination syndromes?

Aléxia G. Pereira, João Pedro C. Filgueiras and Loreta B. Freitas

Supplementary Material

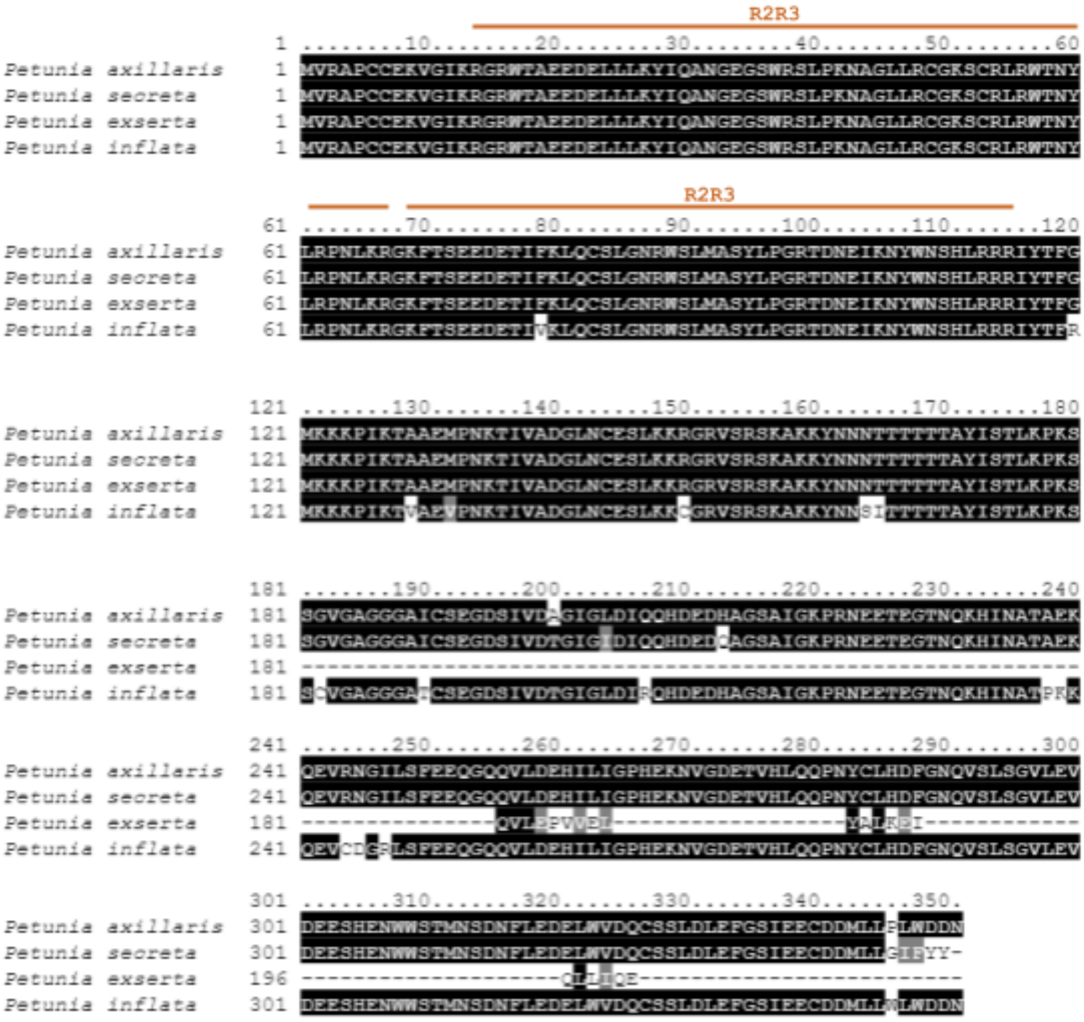

Figure S1: Protein box shading for MYB-FL alignment. The *Petunia exserta* sequence is incomplete due to a mutation that causes a frameshift. The orange lines mark the R2R3 protein domain. Dark boxes highlight conserved amino acids in the sequences.

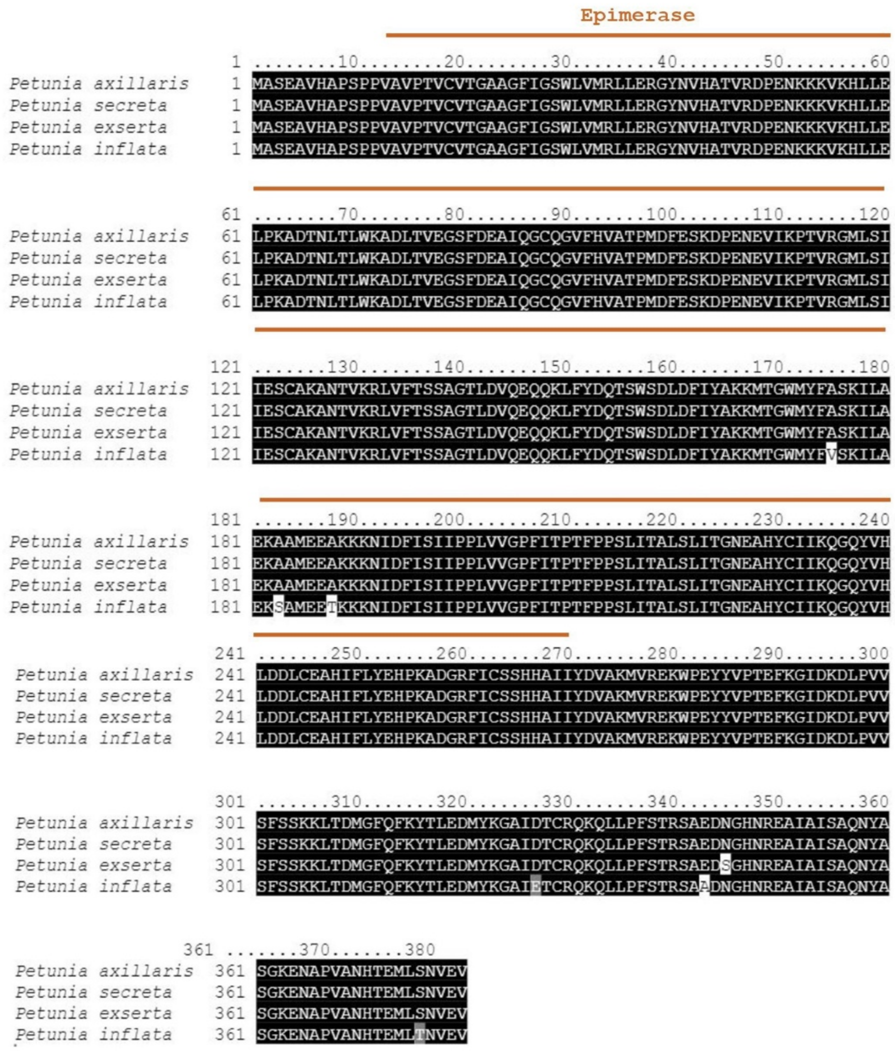

Figure S2: Protein box shading for DFR alignment. All sequences are conserved across species. The orange lines indicate the epimerase protein domain.

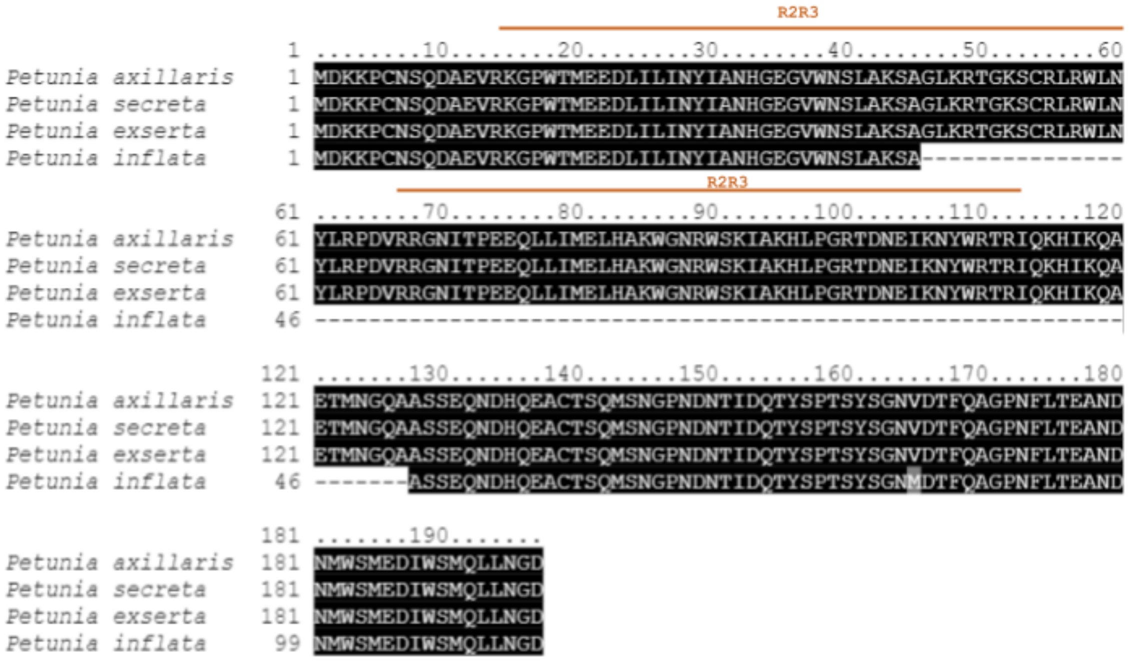

Figure S3: Protein box shading for EOBII alignment. All sequences are conserved across species. The orange lines indicate the R2R3 protein domain.

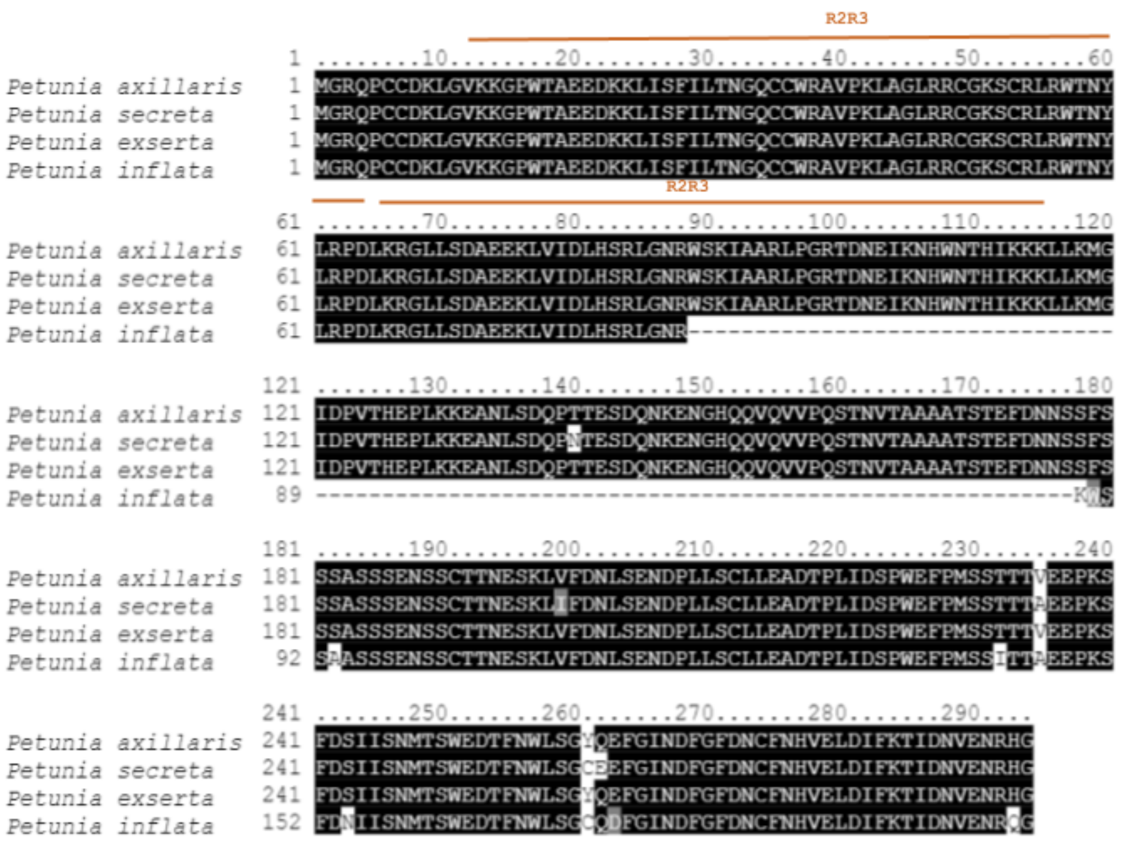

Figure S4: Protein box shading for ODO1 alignment. The orange lines indicate the R2R3 protein domain. Dark boxes highlight conserved amino acids in the sequences.

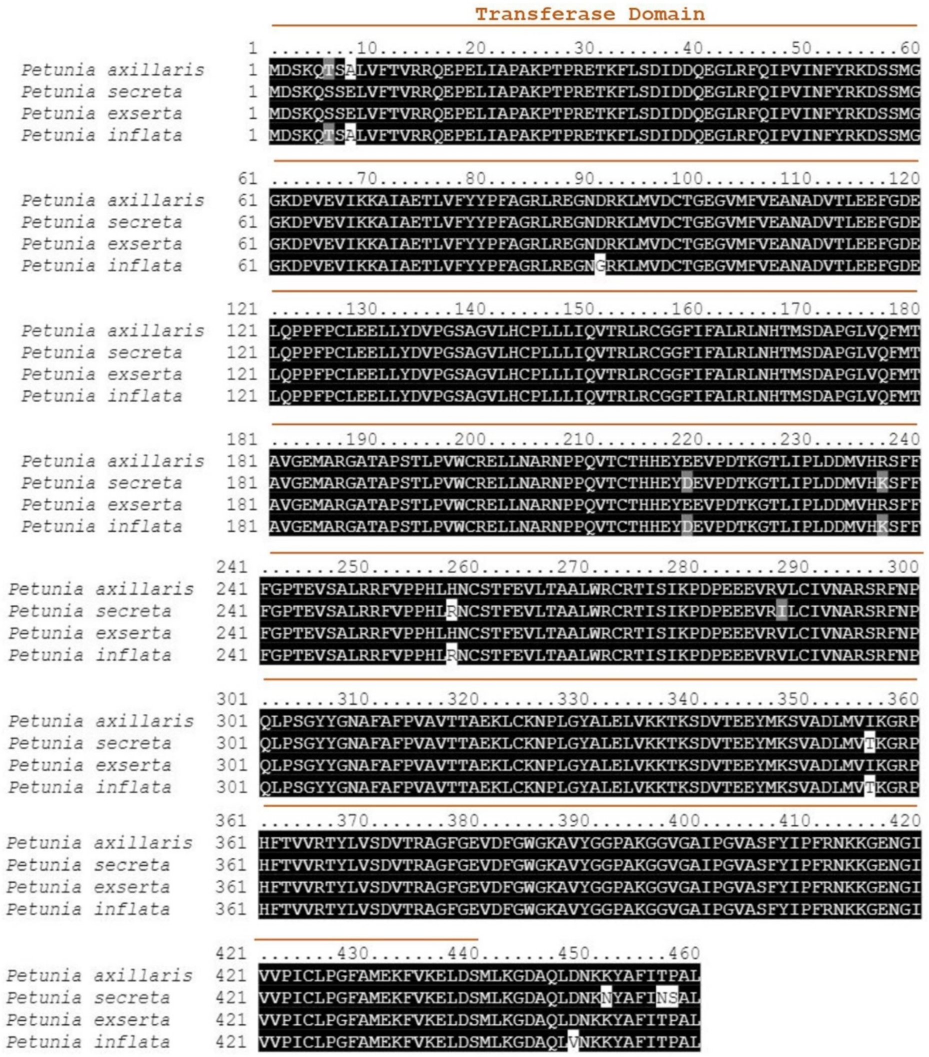

Figure S5: Protein box shading for BPBT alignment. The orange lines indicate the transferase protein domain. Dark boxes highlight conserved amino acids in the sequences.

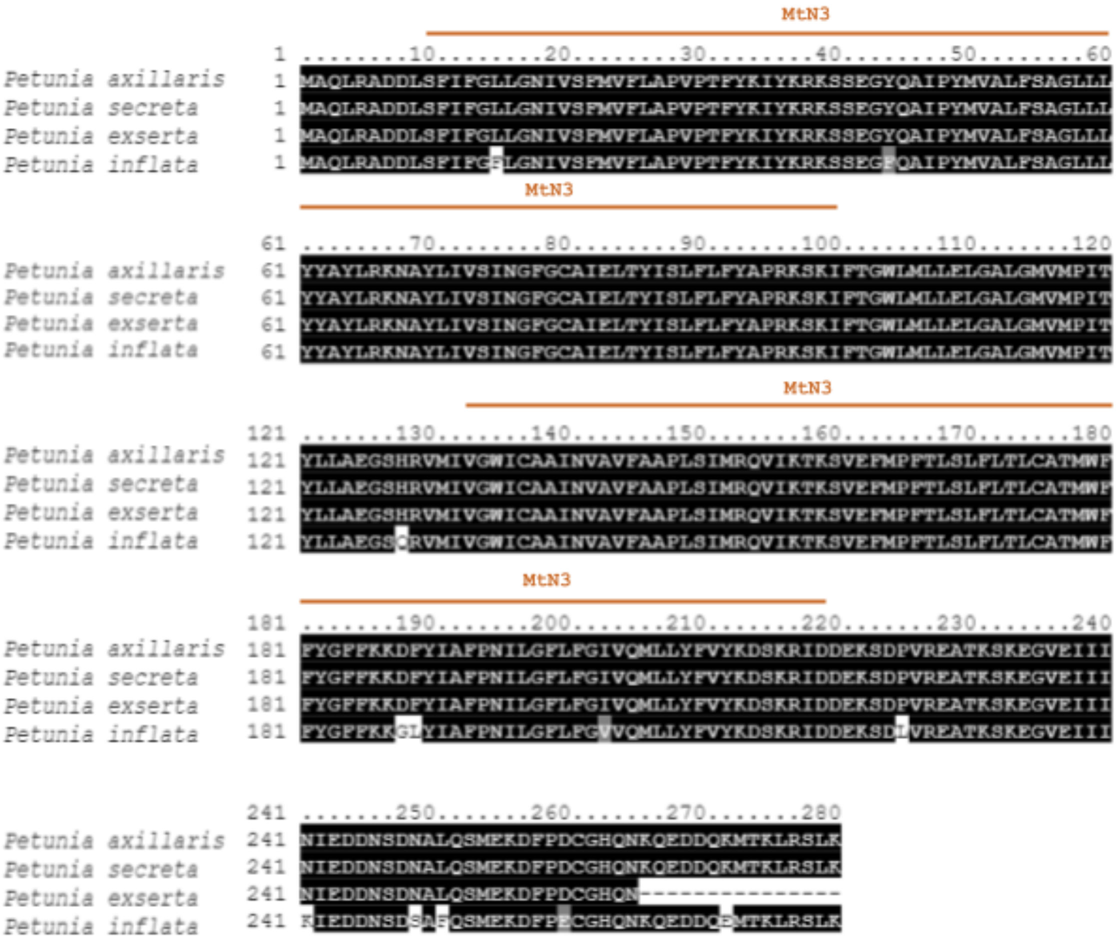

Figure S6: Protein box shading for NEC1 alignment. The orange lines indicate the MtN3 protein domain. Dark boxes highlight conserved amino acids in the sequences.

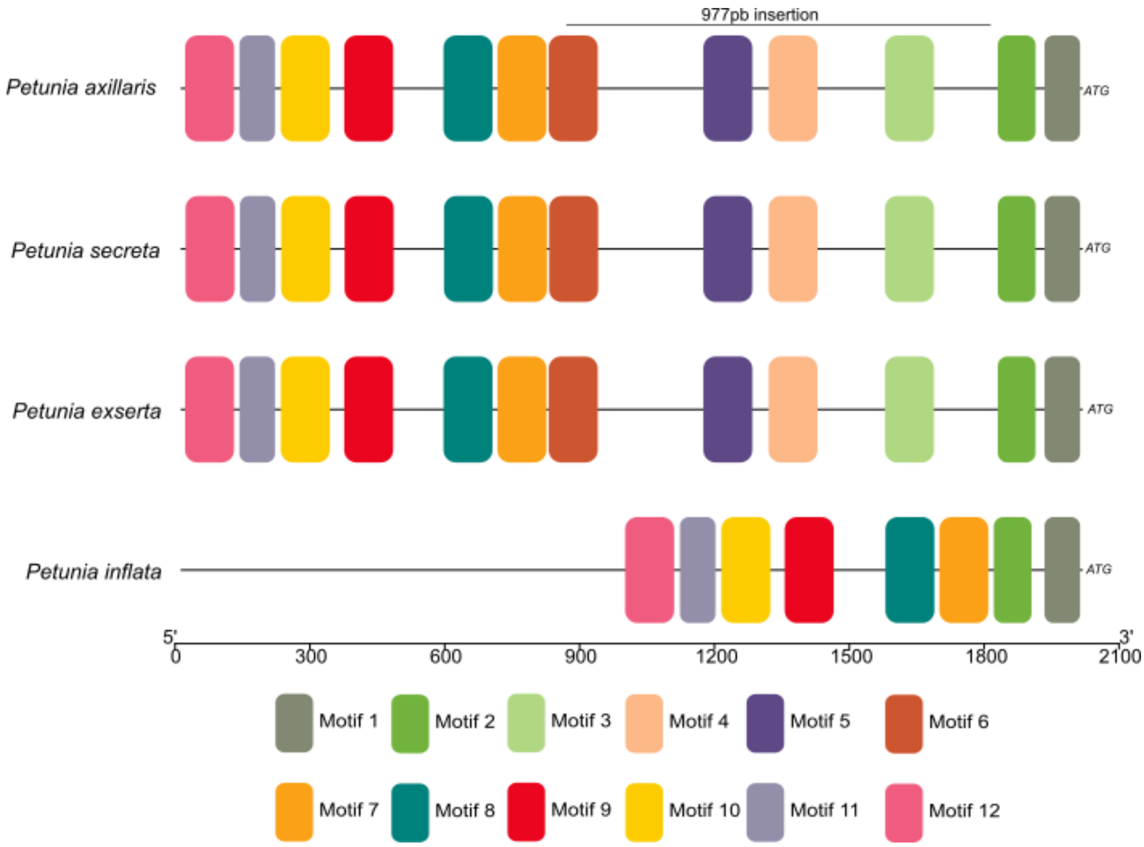

Figure S7: MYB-FL promoter conserved motifs identified through MEME analysis. Motifs are numbered sequentially, starting from the 3′ ATG. Colors denote conserved motifs; gray lines represent non-conserved sequences. The motif length indicates the sequence size.

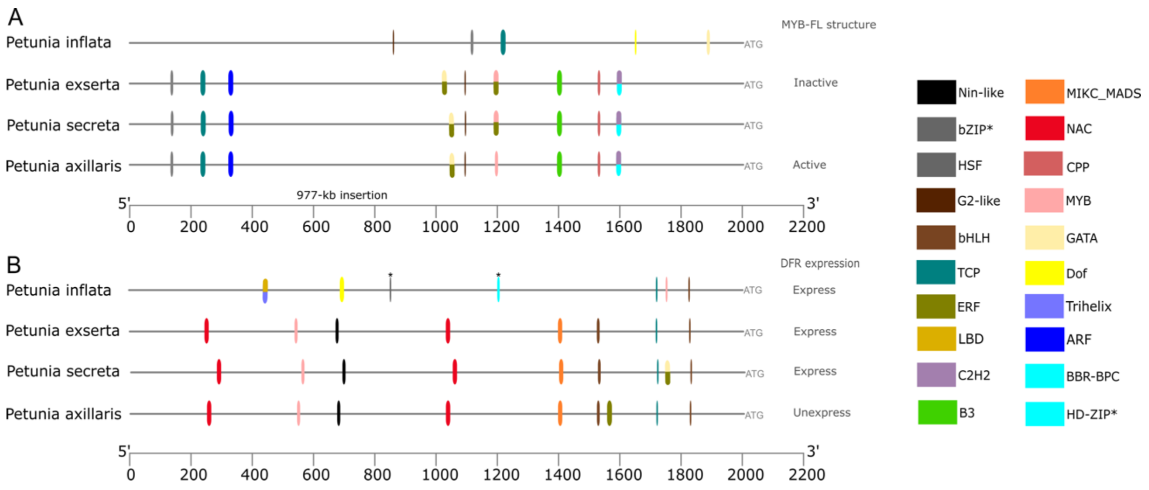

Figure S8: TF-binding sites identified in the promoter regions of the color pathway genes MYB-FL (A) and DFR (B), based on PlantRegMap analysis referencing *Solanum lycopersicum*. Each color represents a transcription factor, as indicated in the legend on the right. Multiple colors indicate overlapping TFs. Asterisks mark sites identified with the same color.

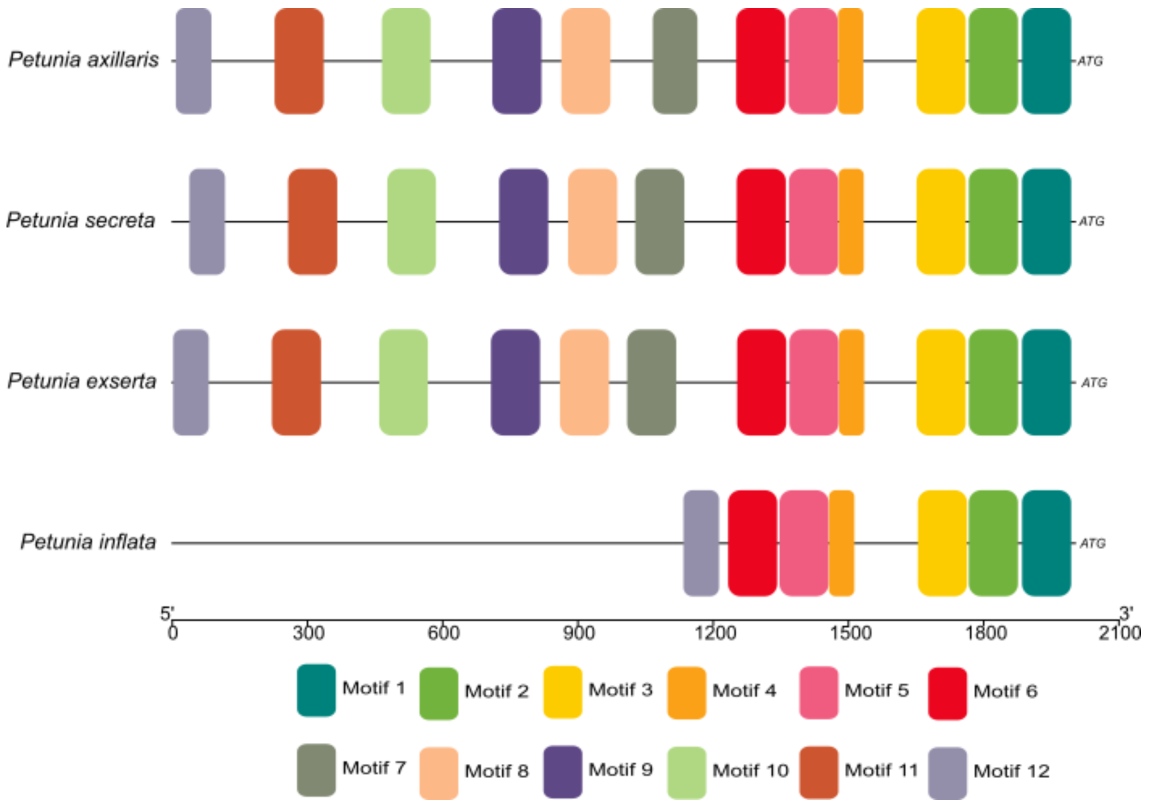

Figure S9: DFR promoter conserved motifs identified through MEME analysis. Motifs are numbered sequentially starting from the 3′ ATG. Colors indicate the conserved motifs; gray lines represent regions that are not conserved. The length of each motif is proportional to its sequence size.

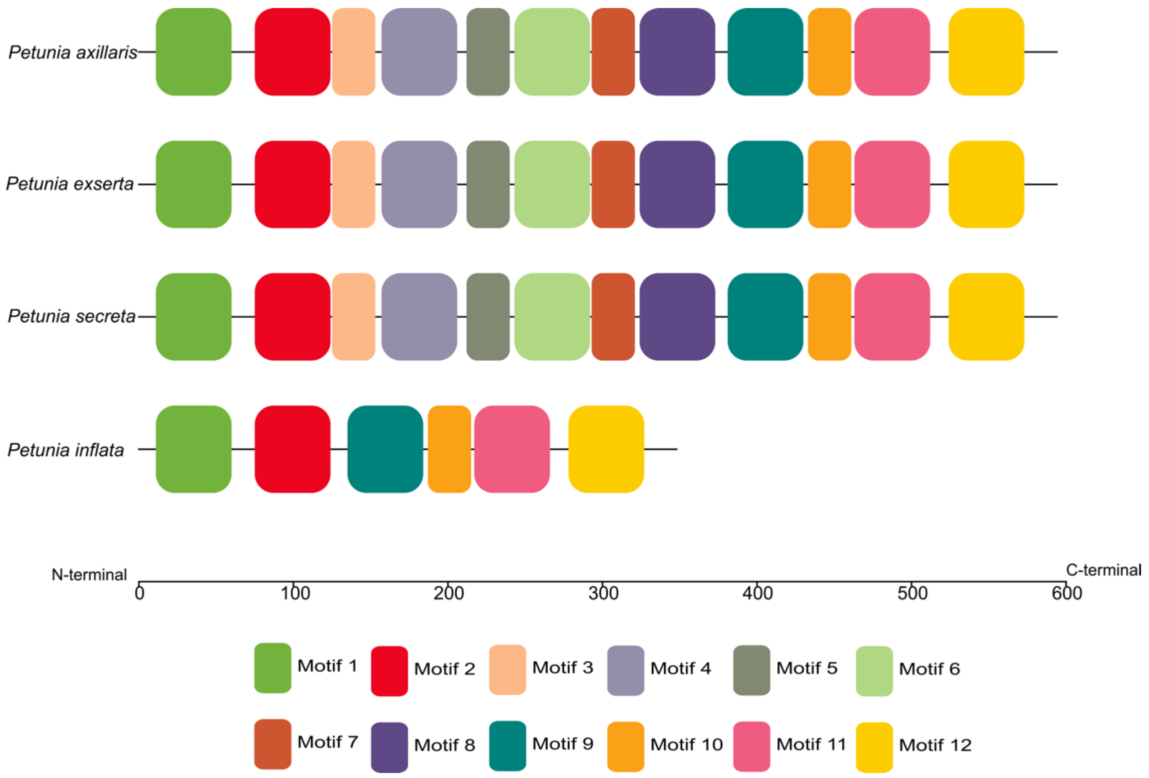

Figure S10: EOBII protein conserved motifs identified in MEME analysis. Motifs are numbered sequentially, starting from the 3’ ATG. Colors represent the conserved motifs, while gray lines indicate non-conserved sequences. The length of each motif corresponds to its sequence size.

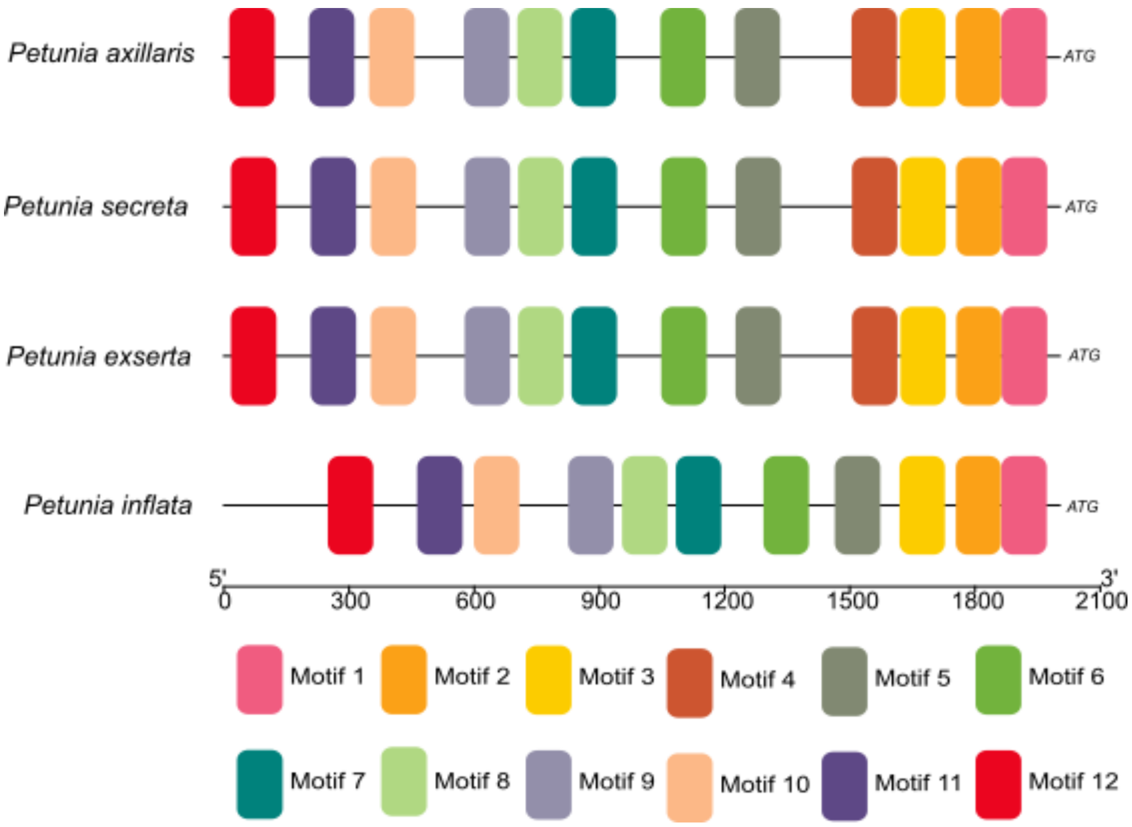

Figure S11: EOBII promoter conserved motifs identified by MEME analysis. Motifs are numbered sequentially, starting from the 3′ ATG. Colors indicate conserved motifs, whereas gray lines show non-conserved sequences. The length of each motif is proportional to its sequence size.

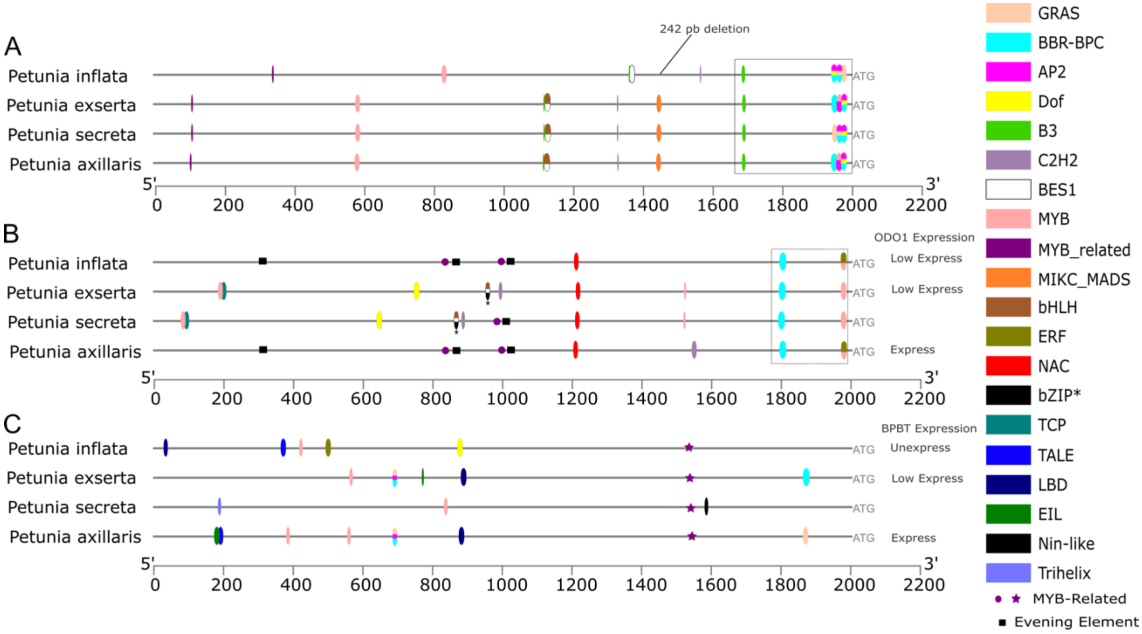

Figure S12: Transcription factor binding sites identified in the promoter regions of the scent pathway (FVBP) genes EOBII (A), ODO1 (B), and BPBT (C), based on PlantRegMap analysis using *Solanum lycopersicum* as the reference. Each color represents a transcription factor, as indicated in the legend on the right. Multiple colors indicate overlapping TFs. Asterisks denote sites identified with the same color. Purple dots mark the binding site of EOBII (AAACCTAAT), while black squares indicate LHY (AAAATATCT) found in the ODO1 promoter using FIMO. Purple stars highlight an ODO1 binding site (CAACAACTAC) observed in the BPBT promoter.

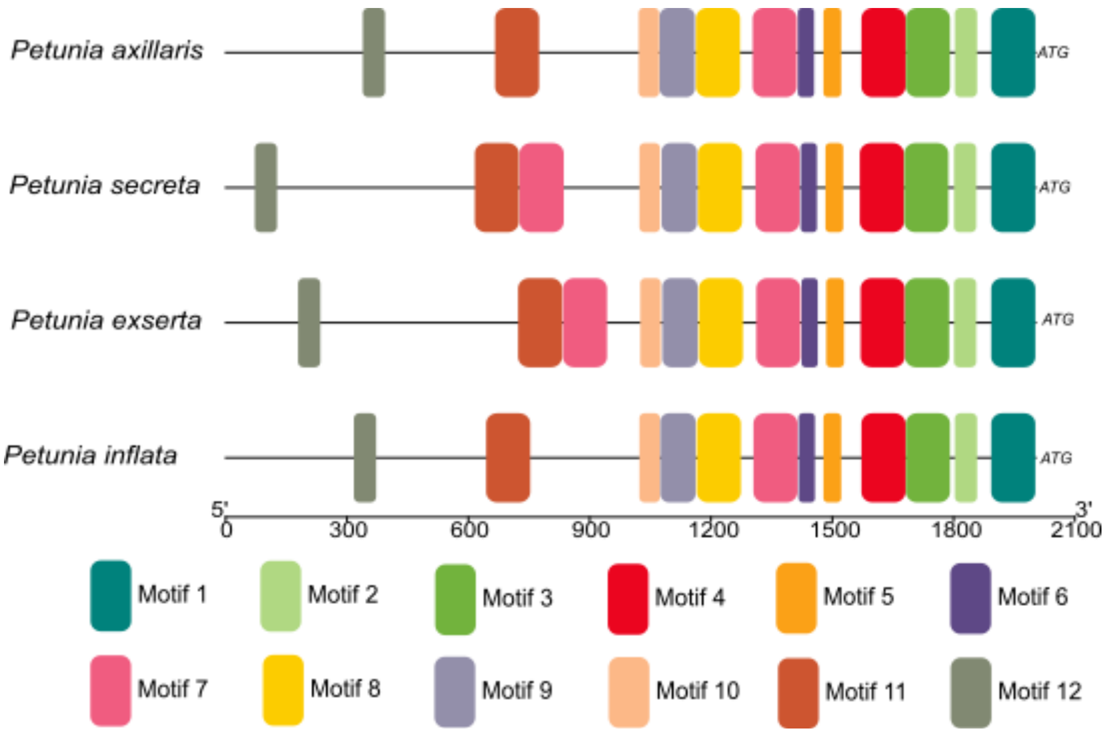

Figure S13: Conserved motifs in the ODO1 promoter identified by MEME analysis. Motifs are numbered sequentially starting from the 3′ ATG. Colors indicate conserved motifs, while gray lines show non-conserved sequences. The length of each motif is proportional to its sequence size.

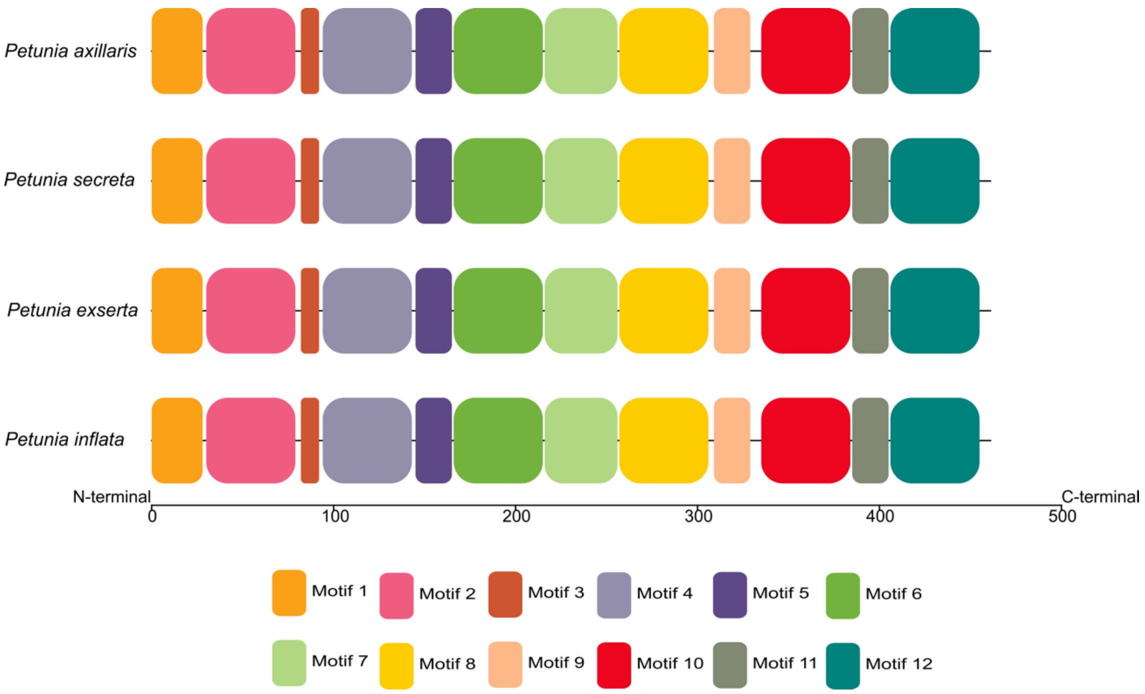

Figure S14: BPBT protein conserved motifs identified by MEME analysis. Motifs are numbered sequentially, starting from the 3′ ATG. Colors represent the conserved motifs, while gray lines indicate non-conserved sequences. The length of each motif is proportional to its sequence size.

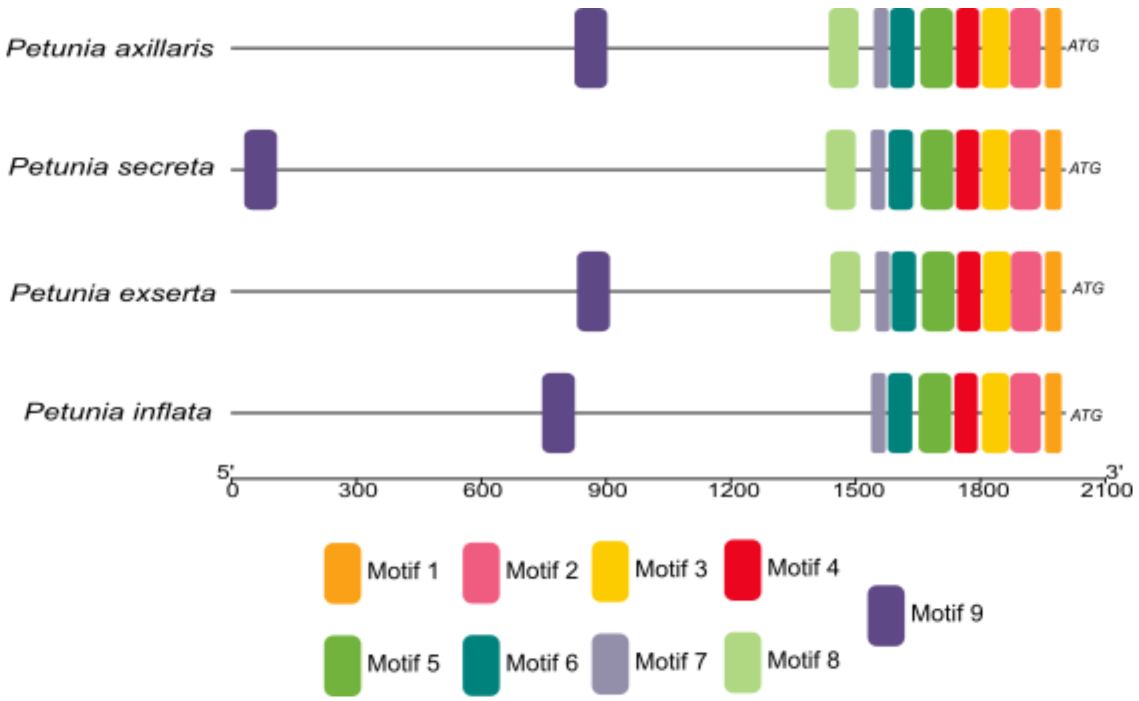

Figure S15: BPBT promoter conserved motifs identified in MEME analysis. Motifs are numbered sequentially, starting from the 3′ ATG. Colors represent conserved motifs; gray lines indicate non-conserved sequences. The length of motifs is proportional to their sequence size.

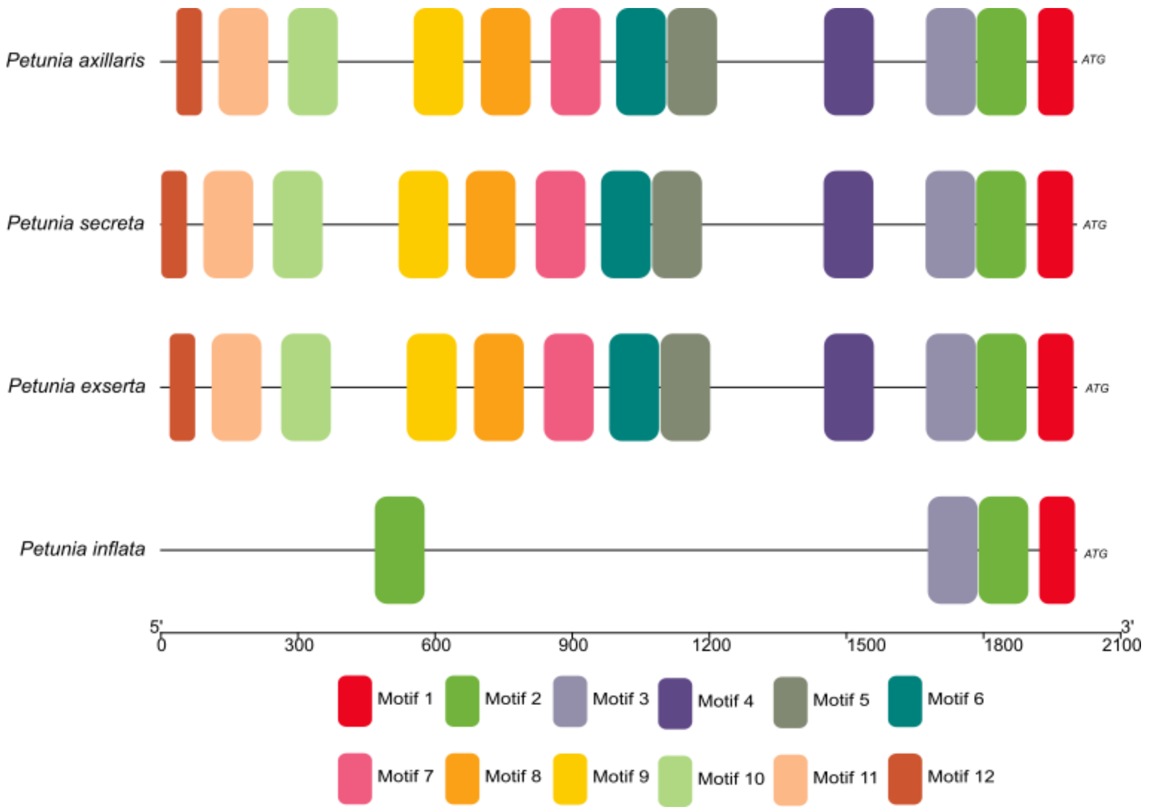

Figure S16: Conserved motifs in the NEC1 promoter identified through MEME analysis. Motifs are numbered sequentially, starting from the 3′ ATG. Colors indicate the conserved motifs; gray lines show non-conserved sequences. The length of each motif is proportional to its sequence size.

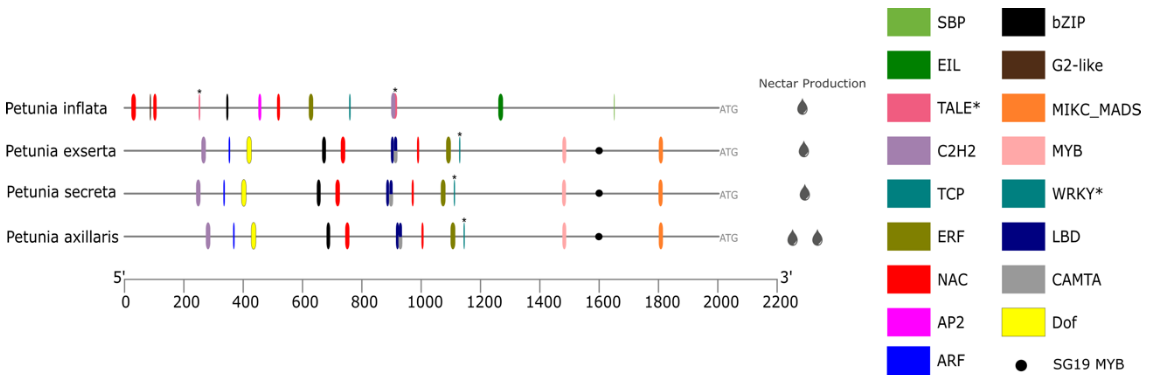

Figure S17: Transcription factor binding sites identified in the promoter regions of the sugar efflux transporter gene NEC1 based on PlantRegMap analysis using *Solanum lycopersicum* as a reference. Each color represents a transcription factor (TF), as indicated in the legend on the right. Multiple colors indicate overlapping TFs. Asterisks denote sites identified with the same color. The black dot indicates the binding site for EOBII (GTTTGGT) as identified with FIMO.

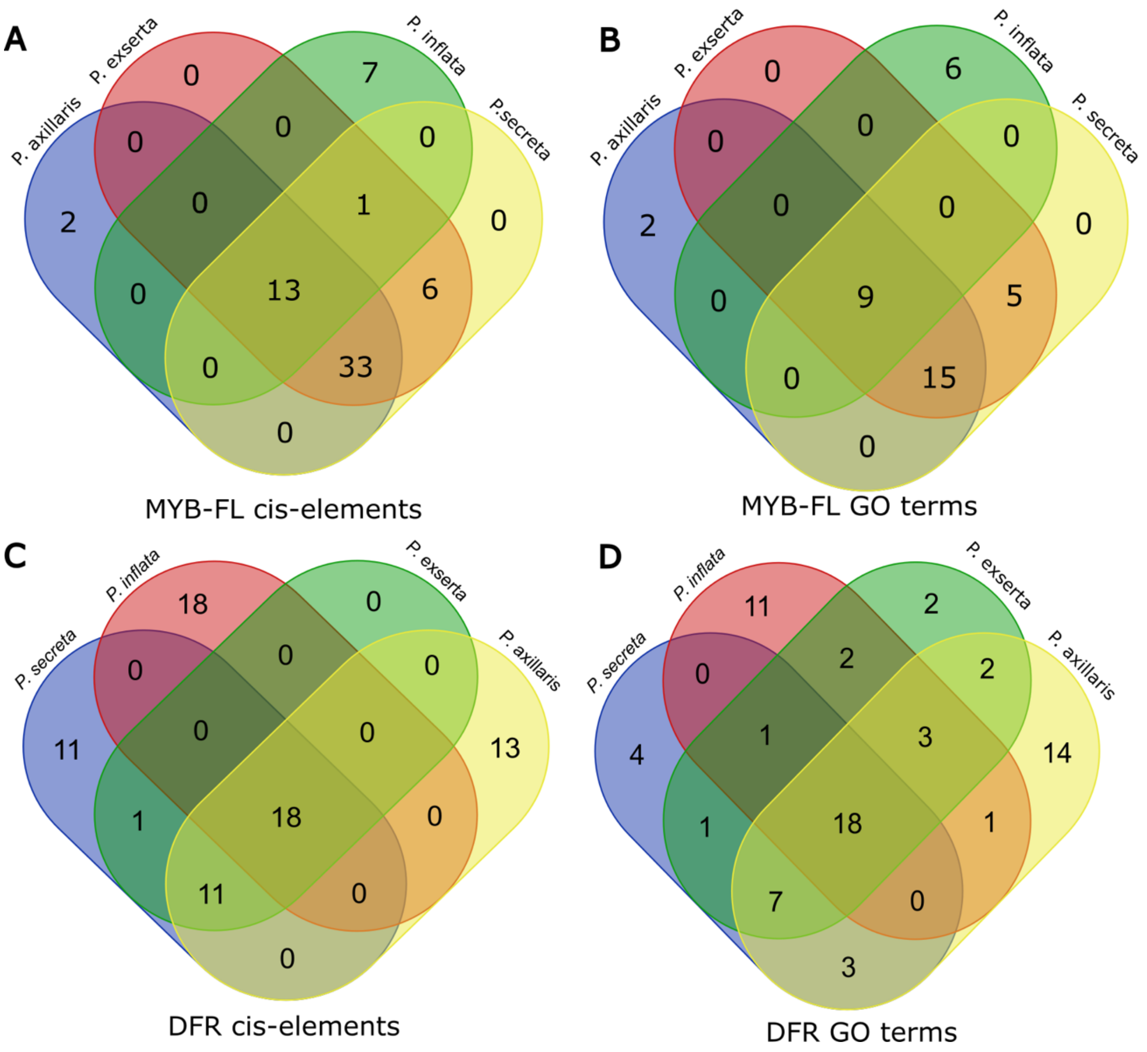

Figure S18: Venn diagrams illustrating the MYB-FL and DFR genes involved in pigment biosynthesis pathways in *Petunia* species. (A and C) - the transcription factors identified in the promoters, emphasizing the number of shared and unique TFs for each *Petunia* species. (B and D) - the same analysis using GO terms, highlighting the number of shared and unique GO terms for each species. This approach connects the number of TFs to the functions performed in each promoter.

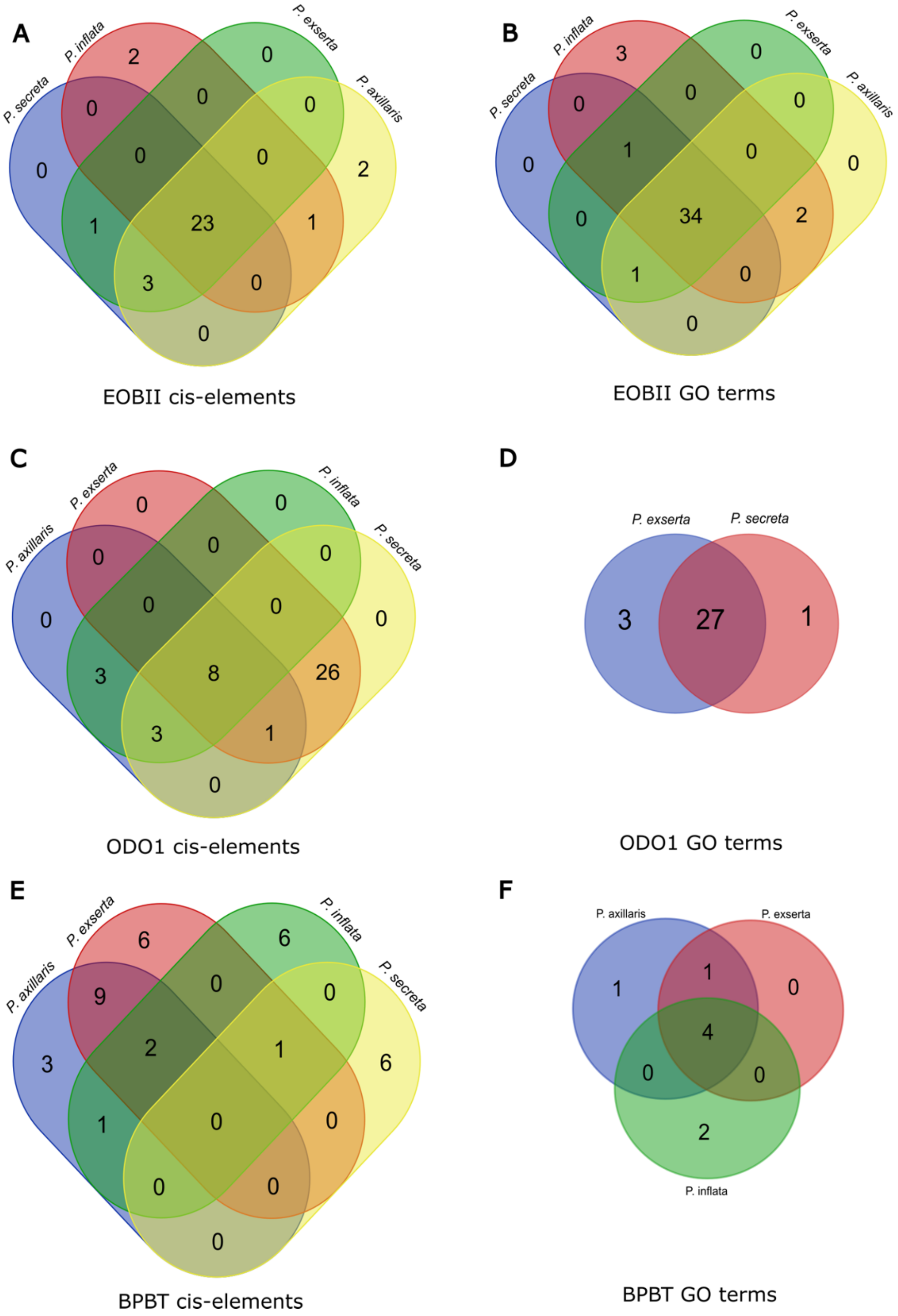

Figure S19: Venn diagrams for the EOBII, ODO1, and BPBT genes involved in volatile biosynthesis pathways in *Petunia* species. (A, C, and E) - the transcription factors found in the promoters, indicating the number of shared and unique TFs for each *Petunia* species. (B, D, and F) - the same analysis with Gene Ontology terms, revealing the number of shared and unique GO terms for each *Petunia* species. This approach highlights the correlation between the number of TFs and the functions performed in each promoter. For ODO1 and BPBT, only species that share GO terms are shown.

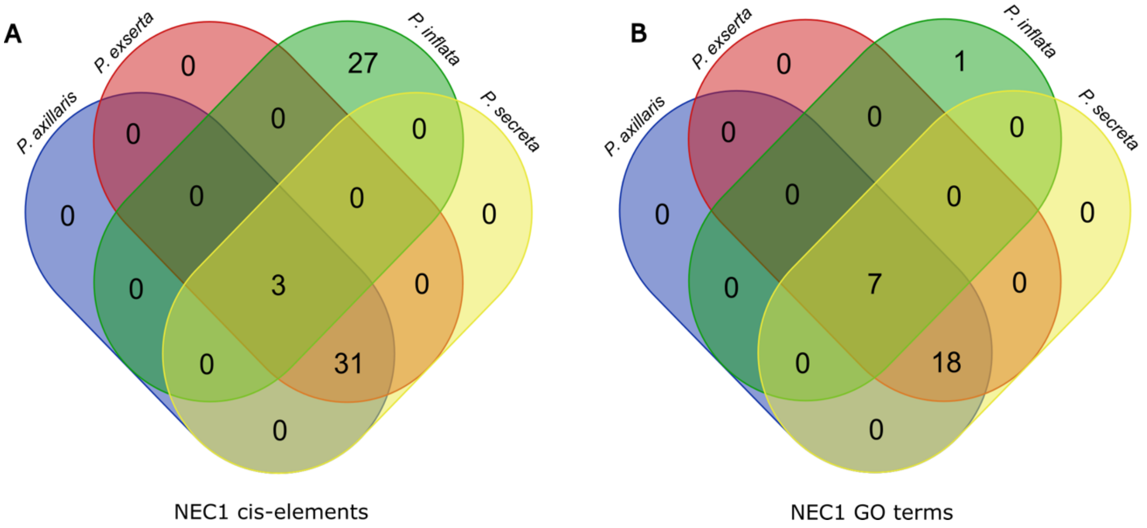

Figure S20: Venn diagrams for the NEC1 gene, which is involved in nectar sugar concentration in *Petunia* species. (A) - the transcription factors identified in the promoters, highlighting the number of shared and unique TFs for each *Petunia* species. (B) - the same analysis performed with Gene Ontology, illustrating the number of shared and unique GO terms for each species. This approach links the number of TFs to the functions associated with each promoter.

Table S1. Genome code for recovered sequences per gene and species

Gene	*Petunia inflata*	*Petunia axillaris*	*Petunia secreta*	*Petunia exserta*
MYB-FL	Peinf101Scf01050: 444864-446866	Peaxi162Scf00886: 213055-215057	JARKIP010000023.1: c4636907-4634905	HiC_scaffold_7: 64137196-64139198
DFR	Peinf101Scf00073: 450163-452165	Peaxi162Scf00366: 656977-658979	JARKIP010000059.1	HiC_scaffold_4: 15759307-15761309
EOBII	Peinf101Scf00394: 1323104-1325106	Peaxi162Scf00080: 601082-603084	JARKIP010000112.1: c1443796-1441794	HiC_scaffold_7: 150854628-150856630
ODO1	Peinf101Scf00284: 55985-57987	Peaxi162Scf00102: 1197140-1199142	JARKIP010000054.1: c5434577-5432575	HiC_scaffold_6: 5195023-5197025
BPBT	Peinf101Scf01180: 116633-118635	Peaxi162Scf00007: 47866-49868	JARKIP010000013.1: c12678013-12676011	HiC_scaffold_7: 18090823-18092825
NEC1	Peinf101Scf00179: 1151044-1153046	Peaxi162Scf00303: 57360-59362	JARKIP010000035.1: c54888518-54886516	HiC_scaffold_6: 73775918-73777920

Table S2. *cis*-Regulatory elements in ODO1, BPBT, and NEC1 genes were revealed using the FIMO tool in the MEME suite.

Gene	Binding Site	Promoter	Species	Strand	Start	End	P-value	q-value	Matched Sequence
EOBII	AACCTAAT	ODO1[1]	*Petunia inflata*	+	882	889	2.18e-05	0.0605	AACCTAAT
EOBII	AACCTAAT	ODO1[1]	*Petunia inflata*	+	995	1002	2.18e-05	0.0605	AACCTAAT
EOBII	AACCTAAT	ODO1[1]	*Petunia axillaris*	+	908	915	2.18e-05	0.0605	AACCTAAT
EOBII	AACCTAAT	ODO1[1]	*Petunia axillaris*	+	1021	1028	2.18e-05	0.0605	AACCTAAT
EOBII	AACCTAAT	ODO1[1]	*Petunia secreta*	+	993	1000	2.18e-05	0.0605	AACCTAAT
LHY	AAWATATCT	ODO1[1]	*Petunia inflata*	+	391	399	1.46e-05	0.0379	AA**T**ATATCT
LHY	AAWATATCT	ODO1[1]	*Petunia inflata*	+	892	900	2.65e-05	0.0493	AA**C**ATATCT
LHY	AAWATATCT	ODO1[1]	*Petunia inflata*	+	1005	1013	1.46e-05	0.0379	AA**A**ATATCT
LHY	AAWATATCT	ODO1[1]	*Petunia axillaris*	+	413	421	1.46e-05	0.0379	AA**T**ATATCT
LHY	AAWATATCT	ODO1[1]	*Petunia axillaris*	+	918	923	2.65e-05	0.0493	AA**C**ATATCT
LHY	AAWATATCT	ODO1[1]	*Petunia axillaris*	+	1003	1011	1.46e-05	0.0379	AA**A**ATATCT
LHY	AAWATATCT	ODO1[1]	*Petunia secreta*	+	1003	1011	1.46e-05	0.0379	AA**A**ATATCT
ODO1	CAACAACTAC	BPBT[2]	*Petunia inflata*	+	1762	1771	1.11e-06	0.0038	CAACAACTAC
ODO1	CAACAACTAC	BPBT[2]	*Petunia inflata*	+	1765	1774	1.11e-06	0.0038	CAACAACTAC
ODO1	CAACAACTAC	BPBT[2]	*Petunia axillaris*	+	1769	1778	1.11e-06	0.0038	CAACAACTAC
ODO1	CAACAACTAC	BPBT[2]	*Petunia secreta*	+	1766	1775	1.11e-06	0.0038	CAACAACTAC
ODO1	CAACAACTAC	BPBT[2]	*Petunia secreta*	+	1769	1778	1.11e-06	0.0038	CAACAACTAC
ODO1	CAACAACTAC	BPBT[2]	*Petunia exserta*	+	1272	1281	1.11e-06	0.0038	CAACAACTAC
ODO1	CAACAACTAC	BPBT[2]	*Petunia exserta*	+	1269	1278	1.11e-06	0.0038	CAACAACTAC
EOBII	GTTAGGT	BPBT[3]	*Petunia inflata*	-	1090	1096	6.51e-05	0.968	GTT**A**GGT
EOBII	GTTAGGT	NEC1[3]	*Petunia axillaris*	+	1607	1613	6.51e-05	0.342	GTT**T**GGT
EOBII	GTTAGGT	NEC1[3]	*Petunia secreta*	+	1608	1614	6.51e-05	0.342	GTT**T**GGT
EOBII	GTTAGGT	NEC1[3]	*Petunia exserta*	+	1608	1614	6.51e-05	0.342	GTT**T**GGT
MYB305	TCACCTAAT	NEC1[4]	*Petunia inflata*	+	1199	1207	8.27e-05	0.174	TCACCTA**TT**
MYB305	TCACCTAAT	NEC1[4]	*Petunia secreta*	+	229	237	8.27e-05	0.174	TCACCTA**AA**
MYB305	TCACCTAAT	NEC1[4]	*Petunia secreta*	+	1481	1489	8.27e-05	0.174	TCACCTA**AA**
MYB305	TCACCTAAT	NEC1[4]	*Petunia axillaris*	+	262	270	8.27e-05	0.174	TCACCTA**AA**
MYB305	TCACCTAAT	NEC1[4]	*Petunia axillaris*	+	1482	1490	8.27e-05	0.174	TCACCTA**AA**
MYB305	TCACCTAAT	NEC1[4]	*Petunia exserta*	+	247	255	8.27e-05	0.174	TCACCTA**AA**
MYB305	TCACCTAAT	NEC1[4]	*Petunia exserta*	+	1482	1490	8.27e-05	0.174	TCACCTA**AA**

References: [1]van Moerkercke *et al.,* 2011; [2]Boersma *et al.,* 2020; [3]Chopy *et al.,* 2023; [4]Liu *et al.,* 2009.
Polymorphic sites are highlighted in bold.

Table S3: Gene ontology analysis per gene and species.

TermID	Name	LogSize	Frequency	Uniqueness	Dispensability	Representative	Value
GO:0000160	phosphorelay signal transduction system	2.394451681	1.24533629	1	0.57225286	null	NaN
GO:0001678	intracellular glucose homeostasis	1.380211242	0.11596249	1	0	null	NaN
GO:0006355	regulation of DNA-templated transcription	3.330819466	10.79459514	1	0.49896227	null	NaN
GO:0007154	cell communication	3.229937686	8.55601492	1	0.08305909	null	NaN
GO:0008150	biological_process	4.297432205	100.00000000	1	0	null	NaN
**GO:0009718**	**anthocyanin-containing compound biosynthetic process**	**1.255272505**	**0.08571140**	**1**	**0**	**null**	**NaN**
GO:0009719	response to endogenous stimulus	3.195068996	7.89553292	1	0.31950938	null	NaN
GO:0009723	response to ethylene	2.442479769	1.39154986	1	0.64176354	null	NaN
GO:0009739	response to gibberellin	2.139879086	0.69073308	1	0.60983412	null	NaN
GO:0009791	post-embryonic development	3.140822180	6.96783301	1	0.64504728	null	NaN
GO:0009888	tissue development	2.800029359	3.17636382	1	0.32676974	null	NaN
GO:0010029	regulation of seed germination	1.954242509	0.44872441	1	0.18345253	null	NaN
GO:0010087	phloem or xylem histogenesis	2.093421685	0.62014722	1	0.40037038	null	NaN
GO:0010262	somatic embryogenesis	1.146128036	0.06554402	1	0.23830656	null	NaN
GO:0010374	stomatal complex development	1.763427994	0.28738530	1	0.61085416	null	NaN
GO:0010440	stomatal lineage progression	1.342422681	0.10587879	1	0.67099158	null	NaN
GO:0014074	response to purine-containing compound	1.041392685	0.05041847	1	0.22113274	null	NaN
GO:0019222	regulation of metabolic process	3.499412126	15.91711203	1	0.47034587	null	NaN
GO:0023052	signaling	3.223495941	8.42996874	1	0.37374755	null	NaN
GO:0031099	regeneration	1.278753601	0.09075325	1	0	null	NaN
GO:0031347	regulation of defense response	2.436162647	1.37138247	1	0.20913372	null	NaN
GO:0032501	multicellular organismal process	3.411788005	13.00796612	1	0	null	NaN
GO:0032502	developmental process	3.448706320	14.16254916	1	0	null	NaN
GO:0033500	carbohydrate homeostasis	1.491361694	0.15125542	1	0.65294432	null	NaN

GO:0033993	response to lipid	2.976808337	4.77462942	1	0.41076444	null	NaN
GO:0035556	intracellular signal transduction	2.709269961	2.57638399	1	0.3287604	null	NaN
GO:0042221	response to chemical	3.448242413	14.14742362	1	0.39465577	null	NaN
GO:0046683	response to organophosphorus	1.041392685	0.05041847	1	0	null	NaN
GO:0048367	shoot system development	2.926856709	4.25531915	1	0.51709003	null	NaN
GO:0048518	positive regulation of biological process	3.015778756	5.22335384	1	0.32904003	null	NaN
GO:0048522	positive regulation of cellular process	2.868056362	3.71584148	1	0.34727433	null	NaN
GO:0048523	negative regulation of cellular process	2.903632516	4.03347787	1	0.27036826	null	NaN
GO:0050789	regulation of biological process	3.705692697	25.59745891	1	0.68804229	null	NaN
GO:0050794	regulation of cellular process	3.660201201	23.05132601	1	0.61349361	null	NaN
GO:0050896	response to stimulus	3.756636108	28.78390642	1	0	null	NaN
GO:0051301	cell division	2.574031268	1.88565090	1	0	null	NaN
GO:0051302	regulation of cell division	1.851258349	0.35292931	1	0	null	NaN
GO:0051716	cellular response to stimulus	3.440121603	13.88524755	1	0.45134688	null	NaN
GO:0065007	biological regulation	3.721728199	26.56045175	1	0	null	NaN
GO:0070542	response to fatty acid	2.334453751	1.08399718	1	0.44379685	null	NaN
GO:0070887	cellular response to chemical stimulus	3.192567453	7.85015630	1	0.65846905	null	NaN
GO:0071326	cellular response to monosaccharide stimulus	1.278753601	0.09075325	1	0.27665159	null	NaN
GO:0071369	cellular response to ethylene stimulus	2.311753861	1.02853686	1	0.61620369	null	NaN
GO:0071395	cellular response to jasmonic acid stimulus	1.959041392	0.45376626	1	0.58400363	null	NaN
GO:0071495	cellular response to endogenous stimulus	2.989449818	4.91580115	1	0.16725481	null	NaN
GO:1901698	response to nitrogen compound	2.071882007	0.58989614	1	0.26387319	null	NaN
GO:1901700	response to oxygen-containing compound	3.188084374	7.76948674	1	0.59997118	null	NaN
GO:1901701	cellular response to oxygen-containing compound	2.829303773	3.39820510	0.676289045	0.59608598	null	NaN
